# The URL1–ROC5–TPL2 transcriptional repressor complex represses the *ACL1* gene to modulate leaf rolling in rice

**DOI:** 10.1093/plphys/kiaa121

**Published:** 2021-01-13

**Authors:** Jingjing Fang, Tingting Guo, Zhiwei Xie, Yan Chun, Jinfeng Zhao, Lixiang Peng, Syed Adeel Zafar, Shoujiang Yuan, Langtao Xiao, Xueyong Li

**Affiliations:** 1 National Key Facility for Crop Gene Resources and Genetic Improvement, Institute of Crop Sciences, Chinese Academy of Agricultural Sciences, Beijing 100081, China; 2 Hunan Provincial Key Laboratory of Phytohormones, Hunan Provincial Key Laboratory for Crop Germplasm Innovation and Utilization, College of Bioscience and Biotechnology, Hunan Agricultural University, Changsha 410128, China; 3 College of Agriculture and Biotechnology, Hunan University of Humanities, Science and Technology, Loudi 417000, China; 4 Shandong Rice Research Institute, Shandong Academy of Agricultural Sciences, Jinan 250100, China

## Abstract

Moderate leaf rolling is beneficial for leaf erectness and compact plant architecture. However, our understanding regarding the molecular mechanisms of leaf rolling is still limited. Here, we characterized a semi-dominant rice (*Oryza sativa* L.) mutant *upward rolled leaf 1* (*Url1*) showing adaxially rolled leaves due to a decrease in the number and size of bulliform cells. Map-based cloning revealed that *URL1* encodes the homeodomain-leucine zipper (HD-Zip) IV family member RICE OUTERMOST CELL-SPECIFIC 8 (ROC8). A single-base substitution in one of the two conserved complementary motifs unique to the 3′-untranslated region of this family enhanced *URL1* mRNA stability and abundance in the *Url1* mutant. URL1 (UPWARD ROLLED LEAF1) contains an ethylene-responsive element binding factor-associated amphiphilic repression motif and functions as a transcriptional repressor via interaction with the TOPLESS co-repressor OsTPL2. Rather than homodimerizing, URL1 heterodimerizes with another HD-ZIP IV member ROC5. URL1 could bind directly to the promoter and suppress the expression of *abaxially curled leaf 1* (*ACL1*), a positive regulator of bulliform cell development. Knockout of *OsTPL2* or *ROC5* or overexpression of *ACL1* in the *Url1* mutant partially suppressed the leaf-rolling phenotype. Our results reveal a regulatory network whereby a transcriptional repression complex composed of URL1, ROC5, and the transcriptional corepressor TPL2 suppresses the expression of the *ACL1* gene, thus modulating bulliform cell development and leaf rolling in rice.

## Teaser

A transcriptional repression complex suppresses the expression of a positive regulator of bulliform cell development, thus modulating bulliform cell development and leaf rolling in rice.

## Introduction

Leaf size and shape are important components of plant architecture (Li et al., 2010). Appropriate leaf rolling maximizes light capture and reduces transpiration under dry conditions (Lang et al., 2004; Moon and Hake, 2011). Bulliform cells are a group of specialized epidermal cells on the adaxial leaf blade surface in monocotyledons that control leaf rolling (Itoh et al., 2005; Xiang et al., 2012). To date, several rice (*Oryza sativa* L.) genes have been identified to regulate abaxial or adaxial leaf rolling by altering the number or size of bulliform cells. Overexpression of *abaxially curled leaf 1* (*ACL1*) increases the number and exaggerates the size of bulliform cells, resulting in abaxially rolled leaves (Li et al., 2010). Similarly, overexpression of the zinc finger homeodomain (HD) class transcription factors *OsZHD1* and *OsZHD2* also induced the abaxial leaf rolling due to the increased number of bulliform cells (Xu et al., 2014). The rice *adaxialized leaf1* (*adl1*) mutant shows abaxially rolled leaves in which large bulliform-like cells are present on either side of leaf (Hibara et al., 2009). In the loss-of-function mutant of *RL14* which encodes a 2OG-Fe oxygenase, altered composition of the secondary cell wall results in water deficiency and shrinkage of bulliform cells leading to incurved leaves (Fang et al., 2012).

The plant-specific HD-leucine zipper (HD-Zip) proteins can be subdivided into four subfamilies: HD-Zip I–IV based on distinct protein sequence features and functions (Elhiti and Stasolla, 2009). There are 16 HD-Zip IV genes in the Arabidopsis genome, of which at least three, namely, *ARABIDOPSIS THALIANA MERISTEM LAYER1* (*ATML1*), *PROTODERMAL FACTOR2* (*PDF2*), and *HOMEODOMAIN GLABROUS11* (*HDG11*), play pivotal roles in differentiation of the epidermis during both embryonic and postembryonic development (Nakamura et al., 2006). Similarly, the maize (*Zea mays*) HD-Zip IV gene *Outer Cell Layer1* (*OCL1*), is critical to specify embryo protoderm identity and maintain the L1 layer of cells in the shoot apical meristem (SAM; Depege-Fargeix et al., 2011). In rice, there are at least nine HD-Zip IV family genes, named *Rice Outermost Cell-specific 1–9* (*ROC1–9*), which exhibit an epidermis-specific expression pattern (Ito et al., 2003). *ROC5* has been further demonstrated to negatively regulate bulliform cell development and modulate leaf rolling (Zou et al., 2011).

The expression of many genes is regulated at the post-transcriptional level through mRNA degradation or stabilization, and the 3′-untranslated region (3′-UTR) of a gene plays an important role in mediating this process (Ross, 1995). Some inherently unstable mRNAs contain a specific motif, that is, the AU-rich elements (ARE; Tavares et al., 2000) in the 3′-UTR. Binding of the ARE-binding proteins elicits rapid degradation of mRNA (Vlasova and Bohjanen, 2008; Simone and Keene, 2013). In addition, the stem-loop domain within a 3′-UTR also modulates gene expression (Holden and Harris, 2004). Potential stem-loop structures are predicted within the 3′-UTR of numerous HD-ZIP IV family members in Arabidopsis, rice, and maize (Javelle et al., 2011; Zalewski et al., 2013; Burgess and Freeling, 2014). There are two evolutionarily conserved motifs of 19 and 21 nucleotides (nt) within the 3′-UTR of *HD-ZIP IV* genes. Importantly, these two motifs are partially complementary, facilitating the formation of a stem-loop structure via base pairing between the two motifs. However, involvement of these two motifs in maintaining proper expression levels of *HD-ZIP IV* family genes has not been verified experimentally.

In this study, we report the *URL1* gene encoding the HD-Zip IV family member ROC8, which regulates leaf rolling in rice. A single nucleotide substitution in one of the two conserved motifs in the 3′-UTR enhances *URL1* mRNA stability in the *Url1* mutant. URL1 interacts physically with ROC5, another HD-Zip IV family member, and TOPLESS (TPL) co-repressor OsTPL2 through its ethylene-responsive element binding factor-associated amphiphilic repression (EAR) motif, which leads to suppressed expression of the downstream gene *ACL1* and inhibited bulliform cell development. These data reveal the important role of *URL1* in leaf shape configuration and the potential role of the two conserved motifs in 3′-UTR in fine-tuning *HD-ZIP IV* mRNA stability.

## Results

### The rice semi-dominant mutant *Url1* shows adaxially rolled leaves due to reduced number and size of bulliform cells

**Figure 1:**
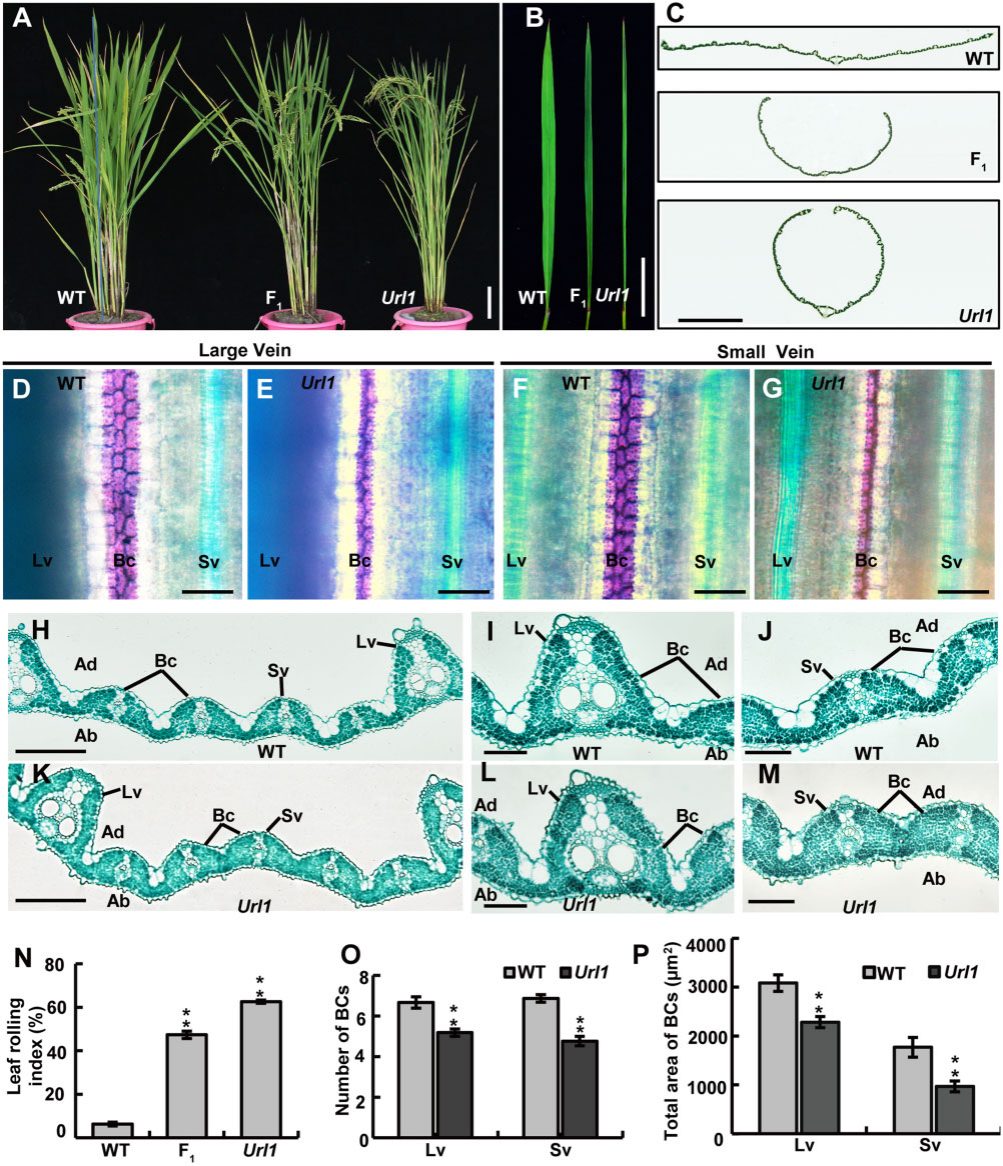
The phenotype of *Url1* mutant. A– C, In mature plants, the leaves rolled adaxially like a cylinder in the *Url1* mutant, semi-rolled in F_1_, compared with the flat leaves in WT. Bars = 10 cm in (A), 5 cm in (B), and 200 µm in (C). D– G, Toluidine blue O staining of bulliform cells which were stained in purple. Bars = 50 *μ*m. H–M, Cross sections of WT and *Url1* mature leaves. Bars = 100 *μ*m in (H) and (K), 50 μm in (I), (J), (L), and (M). Ad, adaxial; Ab, abaxial; Lv, large vein; Sv, small vein; Bc, bulliform cell; N, LRI of WT and *Url1* are shown. Data are presented as mean ± se (*n *=* *15). Significance of data is tested by Student’s *t* test (^**^P <* *0.01). O and P, Bulliform cells number (O) and area (P) of WT and *Url1* are shown*.* Data are presented as mean ± se (*n = *10)*.* Significance of data is tested by Student’s *t* test (^**^P <* *0.01).

To study the underlying mechanisms of rice leaf rolling, we screened the ethyl methanesulphonate-induced mutant population of a *japonica* variety Nipponbare and identified the *Url1* mutant with visibly incurved leaves. The *Url1* mutant had adaxially rolled leaves from the seedling stage, which became more obvious as the rice plants developed (Figure 1). The F_1_ plants derived from a cross between *Url1* and wild type (WT) had semi-rolled leaves, an intermediate between the flat and fully rolled leaves of the homozygous parental plants (Figure 1, A and C). The leaf-rolling index (LRI) was used to quantify the extent of leaf rolling. While the LRI of WT leaves was close to 0, the LRI values were 63% ± 2.72% in the *Url1* mutant and 39% ± 5.23% in the F_1_ heterozygote (Figure 1N). In the F_2_ population, plants with flat, semi-rolled, and rolled leaves had a segregation ratio of 1:2:1 (32:50:25, *χ*^2^ = 1.37, *P *>* *0.05), indicating that the rolled leaf character of *Url1* is caused by a semi-dominant mutation.

Further, bulliform cells of the leaf from 50-d-old plants were stained using toluidine blue O (TBO; Dudley and Poethig, 1993; Hernandez et al., 1999; Li et al., 2010). Bulliform cells stained purple were arranged in three columns along the large vein and small vein in WT. In contrast, only two columns were observed in the *Url1* mutant, although the linear patterning of bulliform cells on the leaf blade did not alter (Figure 1, D–G). To observe bulliform cells and other cell types more clearly, paraffin cross-sectioning of mature leaves was undertaken. Typically, bulliform cells were arranged in groups of 6.67 ± 1.18 adjacent cells near the large veins and 6.88 ± 0.72 near the small veins in WT, whereas the number was significantly reduced to 5.19 ± 0.75 and 4.77 ± 0.83, respectively, in the *Url1* mutant (Figure 1O). Furthermore, the size of bulliform cells was also reduced. The total area of bulliform cells adjoining large veins was reduced by 42.63% in *Url1* (1,768.44 ± 611.25 μm^2^) compared with that in WT (3,082.83 ± 588.17 μm^2^), and reduced by 57.66% for those neighboring small veins (966.66 ± 386.76 μm^2^ in *Url1* versus 2283 ± 377.43 μm^2^ in WT; Figure 1P). Other cell types, such as sclerenchymatous cells at both sides of the large and small veins remained normal in *Url1* (Supplemental Figure S1). Thus, both the number and size of bulliform cells were reduced in the *Url1* mutant, which is responsible for the adaxial leaf rolling.

### *URL1* encodes the HD-ZIP class IV transcription factor ROC8

**Figure 2:**
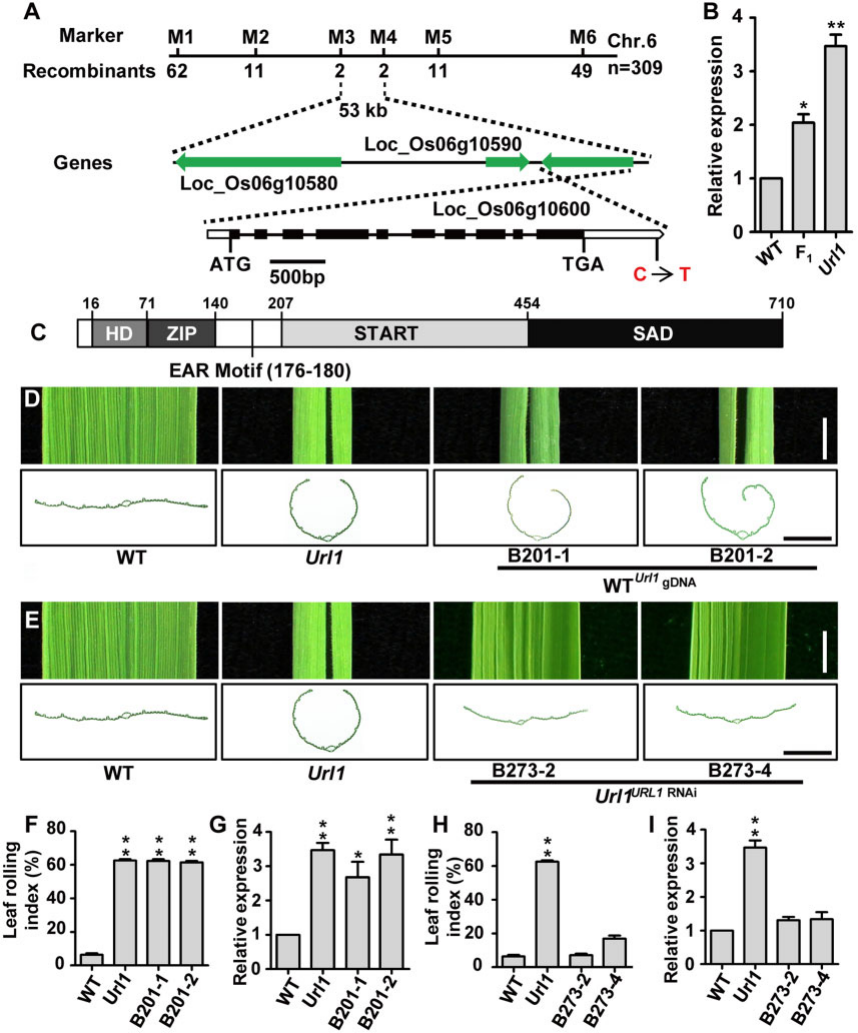
Positional cloning of the *URL1* gene. A, Fine mapping of the *URL1* gene. The molecular markers and numbers of recombinants are indicated above and below the filled bars, respectively. In the *URL1* gene structure, black boxes represent exons; the lines between two black boxes show introns. White arrow indicates the 3′-UTR. B, Reverse transcription quantitative PCR (RT-qPCR) analysis of the *URL1* in 7-d-old seedlings of WT and *Url1*. Data are presented as mean ± se (*n = *3). Significance of data is tested by Student’s *t* test (*P <* *0.05, **P <* *0.01). C, Domain arrangements of the URL1 protein comprised of 710 amino acids. D, Leaf phenotype of complementation lines B201-1 and B201-2 (WT plants transformed with *URL1* genomic fragment amplified from the *Url1* mutant). Bar = 0.3 cm. E, Leaf phenotype of RNAi lines B273-2 and B273-4 (the *Url1* plants transformed with an RNAi construct targeting 3′-UTR of *URL1*). Bar = 0.3 cm. F–I, Quantitative analysis of transgenic plants. LRI was measured in over 13 independent plants in (F) and (H). RT-qPCR analyses of the *URL1* transcript were conducted with three biological replicates in (G) and (I). Significance of data is tested by Student’s *t* test (*P <* *0.05, **P <* *0.01).

The *URL1* locus was primarily mapped between two InDel markers M1 and M6 on the short arm of chromosome 6 and further narrowed to a 53-kb region delimited by two markers M3 and M4 (Figure 2A). Three genes were annotated within this region by the Rice Genome Annotation Project (http://rice.plantbiology.msu.edu/). Sequence comparison revealed a C-to-T substitution at the 679th nt after the stop codon of *LOC_Os06g10600* (Figure 2A). Using the 3′- and 5′-rapid amplification of cDNA ends (RACE) technique, full-length cDNA sequence of *LOC_Os06g10600* was determined, which has a long 3′-UTR of 732 nt. Therefore, the mutation site is located within the 3′-UTR of *LOC_Os06g10600*. As the 3′-UTR plays an important role in the post-transcriptional regulation of gene expression (Ross, 1995), we examined the expression level of *LOC_Os06g10600*. The transcript level of *LOC_Os06g10600* increased significantly in *Url1* leaves and also moderately in the F_1_ heterozygote (Figure 2B), consistent with the semi-dominant inheritance pattern. Therefore, *LOC_Os06g10600* is designated as *URL1* hereafter.

The *URL1* gene consists of 10 exons and 9 introns (Figure 2A), encoding the 710-residue HD-ZIP IV family transcription factor ROC8 (Supplemental Figure S2). URL1 contains four typical domains of HD-ZIP IV proteins: an N-terminal HD, followed by a leucine-zipper motif (ZIP), a steroidogenic acute regulatory protein-related lipid transfer (START) domain, and a START-adjacent domain at the C-terminus (Figure 2C). Phylogenetic analysis showed that URL1 is the closest to Arabidopsis HOMEODOMAIN GLABROUS11 (HDG11) and HDG12, maize Outer Cell Layer15 (OCL15) and OCL17, and rice RICE OUTERMOST CELL-SPECIFIC6 (ROC6; Supplemental Figure S3).

### Increased expression of *URL1* is responsible for the rolled leaves phenotype in *Url1*

To verify whether the point mutation in the 3′-UTR of *URL1* is responsible for the *Url1* phenotype, a 6,070-bp genomic DNA fragment of the *URL1* gene from the *Url1* mutant was introduced into WT. The transgenic lines obtained displayed the adaxially rolled leaf phenotype with increased *URL1* transcript level, mimicking the leaf-rolling phenotype of the *Url1* mutant (Figure 2, D, F, and G). Additionally, the RNAi construct targeting the 3′-UTR of *URL1* was introduced into the *Url1* mutant. As expected, reducing *URL1* expression in the *Url1* mutant markedly suppressed the adaxial rolling of leaf in the transgenic lines (Figure 2, E, H, and I). Furthermore, we generated *URL1* knockout mutants through the CRISPR-Cas9 approach in the *Url1* mutant background (Ma et al., 2015). These *URL1* loss-of-function mutants exhibited abaxially rolled leaves with increased number and size of bulliform cells (Supplemental Figure S4). Therefore, the phenotypes of both gain- and loss-of-function mutants collectively indicated that URL1 is a key regulator of leaf rolling via bulliform cell development control.

### Mutation in the conserved motif in 3′-UTR enhances the stability of *URL1* mRNA

**Figure 3:**
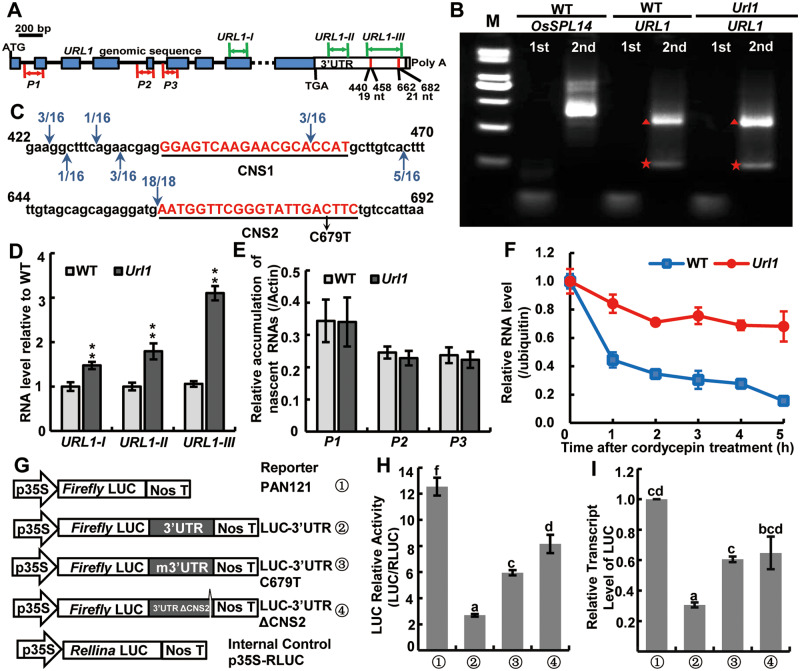
A single-base substitution in the conserved complementary motifs unique to 3′-UTR of this family enhanced the *URL1* mRNA stability. A, Position of the two conserved motifs CNS1 (19 nt) and CNS2 (21 nt) in the 3′-UTR of *URL1*. URL1-I, URL1-II, and URL1-III indicate the locations of primer sets used in (D). P1, P2, and P3 indicate the locations of primer sets used in (E). B, 5′-RLM-RACE showed that two putative cleavage products of about 300 and 100 bp (red triangle and pentagram) were present in both WT and *Url1*. Amplification of miRNA156 cleavage product of *OsSPL14* was used as a positive control. C, The cleavage sites detected within 3′-UTR of *URL1*. Sixteen and 18 independent clones were sequenced for the large and small cleavage product, respectively. Arrows indicate cleavage sites relative to the CNS1 or CNS2 motif. D, RT-qPCR analysis of the *URL1* mRNA transcript level in the *Url1* mutant and WT using primer sets URL1-I, URL1-II, and URL1-III located in different regions of *URL1*, respectively. The rice *ubiquitin* gene was used as an internal control and the *URL1* expression level in the *Url1* mutant was normalized to the WT. Data are presented as mean ± se (*n *=* *4). Significance of data is tested by Student’s *t* test (**P <* *0.01). E, Quantification of nascent *URL1* transcrips by biotin-16-UTP immunocapture nuclear run-on RT-qPCR in WT and *Url1*. RT-qPCR analysis were done with three primer sets P1, P2, and P3 which span the first, fourth, and fifth intron–exon bondary, respectively. F, The *URL1* mRNA decay assay in the *Url1* mutant and WT. *URL1 m*RNA level was determined by RT-qPCR analysis after treatment with cordycepin for 0, 1, 2, 3, 4, and 5 h. *URL1* expression levels before cordycepin treatment (0 h) in WT and the *Url1* mutant are set as 1.0, respectively. Data are presented as means ± se (*n *=* *5). G, Schematic representation of 3′*-*UTR constructs used in the luciferase assay. H and I, Relative luciferase activities (H) and mRNA level (I) were measured when 3′*-*UTR*, m*3′*-*UTR, Δ3′*-*UTR of *URL1* were inserted into the *P35S::LUC* reporter vector*.* Data are presented as mean ± se (*n *=* *4), and the same letters above each bar indicate that means did not differ significantly at the 0.05 level in Tukey’s multiple comparison test.

Next, we aimed to explain why the point mutation in the 3′-UTR of *URL1* results in increased transcript level. Like most members of the HD-ZIP IV family, the 3′-UTR of *URL1* harbors two evolutionarily conserved motifs, i.e., CNS1 (conserved noncoding sequence1) and CNS2 (Javelle et al., 2011; Burgess and Freeling, 2014). The C-to-T substitution at the 679th nucleotide after the stop codon resides within the CNS2 motif (Figure 3, A and C). CNS1 and CNS2 are partially complementary to each other and the base pairing between them mediates the formation of a stem-loop structure as predicted by the software RNAfold (Gruber et al., 2008). The C679T substitution changed the G–C base pairing to G–U mismatch, which may reduce the stability of stem-loop structure (Supplemental Figure S5).

As the stem-loop structure in the 3′-UTR has been characterized as an mRNA-destabilizing element (Paschoud et al., 2006; Mullen and Marzluff, 2008; Leppek et al., 2013; Chang et al., 2016), we examined potential cleavage sites in the 3′-UTR of *URL1*. Using the RNA ligase-mediated 5′-rapid amplification of cDNA ends (RLM-5′RACE) technique, two fragments of about 300 and 100 bp were obtained (Figure 3B). Amplification of the cleaved fragment of *OsSPL14* by miRNA156 was used as a positive control (Jiao et al., 2010). Cloning and sequence analysis of the 300-bp fragment revealed six cleavage sites within or surrounding the CNS1 motif. Analysis of the 100-bp fragment revealed one cleavage site at the 5′-boundary of CNS2 (Figure 3C). To detect the effects of cleavage sites on the *URL1* mRNA stability, we designed three primer sets located within the CDS region (URL1-I), 3′-UTR region upstream of CNS1 (URL1-II), and 3′-UTR region encompassing both CNS1 and CNS2 (URL1-III) to perform reverse transcription quantitative PCR (RT-qPCR) analysis (Figure 3A). All three primer sets detected a significant increase in the *URL1* expression in the *Url1* mutant, wherein URL1-III flanking the cleavage sites detected the most pronounced increase (Figure 3D). To explore the possible mechanisms of *URL1* mRNA accumulation in the *Url1* mutant, we first performed a nuclear run-on RT-qPCR (Folta and Kaufman, 2006; Patrone et al., 2000; Roberts et al., 2015) to quantify the nascent transcripts of *URL1* in the *Url1* mutant and WT using the three primer sets P1, P2, and P3 spanning the first, fourth, and fifth intron–exon boundary, respectively (Figure 3A). The results showed that there was no significant difference in nascent transcription between WT and the *Url1* mutant (Figure 3E), suggesting that the C679T mutation did not affect nascent transcription and that *URL1* mRNA levels may be modulated post-transcriptionally. We then compared the decay rate of the WT and mutant *URL1* mRNA using RT-qPCR. Upon treatment with cordycepin, which inhibits transcription (Hori and Watanabe, 2008), the *URL1* transcript level was detected at 1-h intervals. The mutated *URL1* (C679T) degraded much slower than the WT *URL1* (Figure 3F), suggesting that the mutant *URL1* mRNA has increased stability. These results confirmed that the stem-loop structure in the 3′-UTR triggers degradation of *URL1* mRNA.

To corroborate that the 3′-UTR of *URL1* was involved in mRNA destabilization, we used a firefly luciferase-based reporter system (Figure 3G; Ruberti et al., 1991). The 3′-UTR of *URL1* from both WT and the *Url1* mutant was transcriptionally fused to the 3′-end of the *LUCIFERASE* (*LUC*) ORF. A fourth reporter construct was also created in which the CNS2 motif was deleted. The four LUC reporter constructs along with the internal control (*Renilla* luciferase, RLUC) were co-transformed into rice leaf protoplasts. Luciferase activity in the cells transformed with *LUC-3*′*-UTR* was significantly lower than that in those transformed with the control *LUC* construct. However, cells transformed with two mutated versions of 3′-UTR (*LUC-3*′*UTRC679T* and *LUC-3*′*UTRΔCNS2*) showed significantly increased luciferase activity compared to those transformed with the intact 3′-UTR (*LUC-3*′*UTR*; Figure 3H). RT-qPCR assay on the same samples revealed that *LUC* mRNA level correlated well with the luciferase activity. The presence of 3′-UTR significantly reduced the *LUC* mRNA level, whereas expression of *LUC-3*′*UTRC679T* and *LUC-3*′*UTRΔCNS2* resulted in partial restoration of *LUC* mRNA level (Figure 3I). These results suggest that the 3′-UTR of *URL1* is responsible for its mRNA stability.

Finally, we confirmed the effect of C679T substitution on *URL1* mRNA stability *in planta*. We generated transgenic rice plants expressing *URL1* cDNA with the WT or mutated 3′-UTR driven by the rice *Actin1* promoter. All 14 transgenic lines of the WT *URL1* cDNA (B197) showed flat-leaf phenotypes in the T_0_ generation. However, 10 out of 12 lines transformed with the mutant *Url1* cDNA (B320) showed adaxial leaf rolling (Supplemental Figure S6A). RT-qPCR analysis of representative lines showed that the *URL1* mRNA level was increased in the B320 lines but not in the B197 lines, consistent with their LRI (Supplemental Figure S6, B and C). These data indicate that the *Url1* mutant mRNA was more readily accumulated than the WT *URL1* mRNA, as a result of the enhanced mRNA stability.

### Expression pattern and subcellular localization of URL1

**Figure 4:**
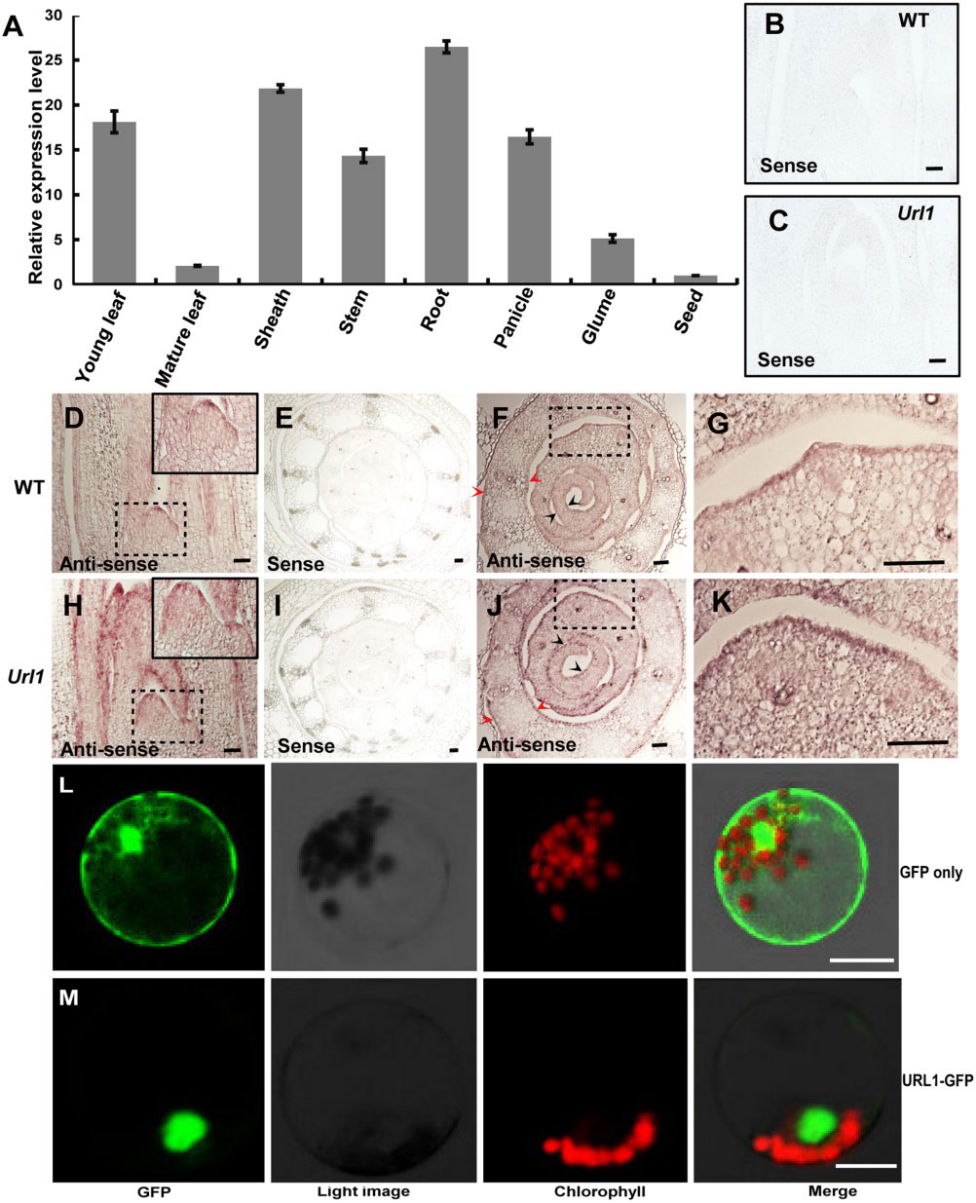
Expression pattern and subcellular localization of the URL1 protein. A, RT-qPCR analysis of *URL1* expression in various tissues including young leaf at the seedling stage, mature leaf at the heading stage, sheath, stem, root, panicle, glume, and seed. Rice *ubiquitin* was used as an internal control. Data are presented as mean ± se (*n *=* *3). B–K, *In situ* hybridization to detect *URL1* transcripts in WT and the *Url1* mutant on the longitudinal section from the shoot apex region of 7-d-old rice seedling (B, C, D, H) and the cross section from young leaves of 7-d-old rice seedling (E, F, G, I, J, K). Black-dotted areas in (D) and (H) were magnified and inserted in the upper right corner, respectively. Black and red arrow heads indicate the *in situ* hybridization signals in the epidermal cells in young leaves and mature leaves, respectively, at both the abaxial and adaxial sides. Black-dotted areas in (F) and (J) were magnified and shown in (G) and (K), respectively. The sense probe was hybridized and used as negative controls in (B, C, E, I). Bars = 200 *μ*m. L and M, Subcellular localization of the URL1 protein. Transient expression of URL1**–**GFP fusion protein in rice protoplasts revealed that URL1 is mainly located in the nucleus*. p35S:GFP* was used as the control. Bars = 20 *μ*m.

RT-qPCR analysis revealed that *URL1* was ubiquitously expressed in all tissues with a relatively higher expression in leaf, root, and stem. In particular, *URL1* expression in younger leaves was significantly higher than that in the older leaves (Figure 4A). Histological staining of the *URL1p*:*GUS* transgenic rice plants revealed strong GUS activity in the epidermal layer of leaf blade and sheath, although signal could also be detected in the inner tissues (Supplemental Figure S7, I and J). In the *in situ* hybridization analysis, *URL1* mRNA was detected in the entire L1 layer of SAM, axillary meristem, leaf primordium, and young leaves (Figure 4, B–D and H; Supplemental Figure S7, L–N). Cross-sectional analysis of the young leaves revealed more intense signal in the epidermal cell layer and vasculature tissue (Figure 4, E–G and I–K). The *URL1* expression pattern in the *Url1* mutant was similar to that in WT. However, the signal intensity was greatly increased in the *Url1* mutant (Figure 4, B–K), which is consistent with the enhanced stability of *Url1* mutant mRNA. Further analysis showed that the URL1::GFP protein localized in the nucleus of rice protoplast (Figure 4, L and M), consistent with the function of HD-ZIP IV family proteins as transcriptional regulators.

### URL1 recruits TPL2 with its EAR motif to function as a transcriptional repressor

**Figure 5:**
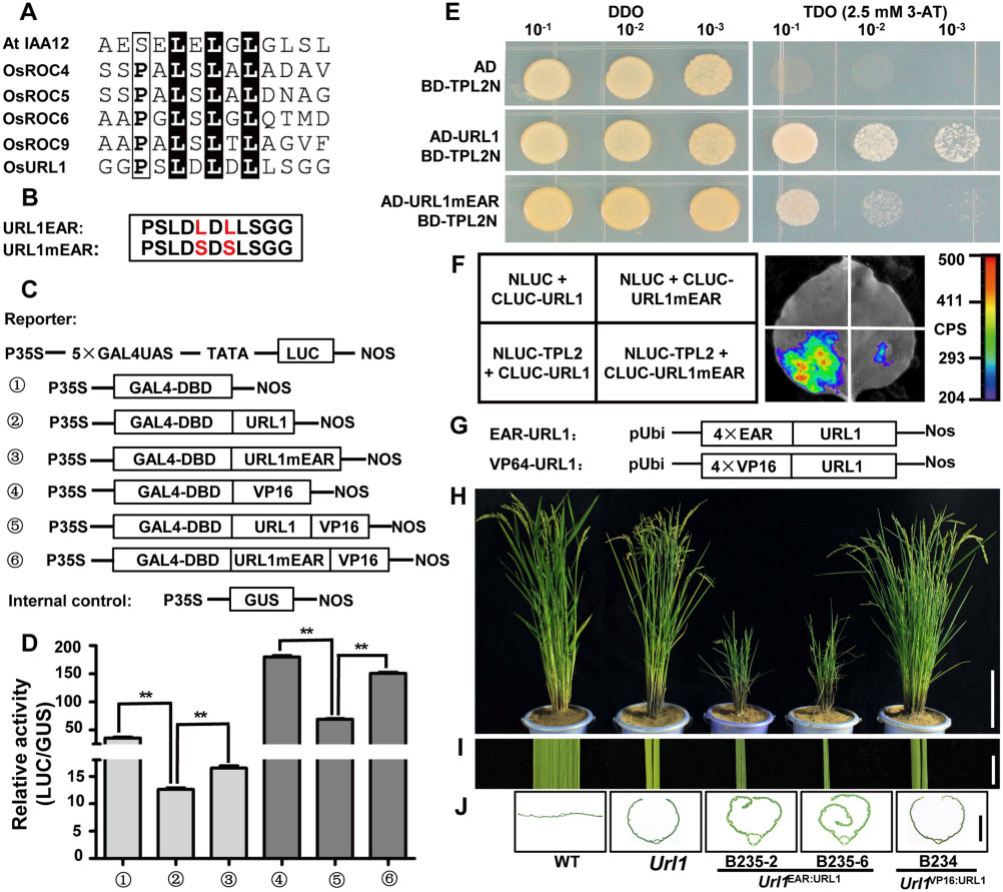
URL1 interacts with TPL2 through the EAR motif and functions as a transcriptional repressor in modulating adaxial rolling of leaf. A, Alignment of the EAR motif from several rice and Arabidopsis proteins*.* B, URL1mEAR in which the last two conserved Leu residues in the EAR motif were replaced by Ser residue. C, Schematic representation of reporter and effector constructs used in the luciferase assay. D, Relative luciferase activities using URL1, URL1mEAR, URL1-VP16, and URL1mEAR-VP16 as an effector compared with the GAL4-DBD and GAL4-VP16 control, respectively. Data are presented as mean ± se (*n *=* *4). Significance of data is tested by Student’s *t* test (**P <* *0.01). E, URL1 interacted with the N-terminus of TPL2 through the EAR motif in yeast two-hybrid assay. Transformed yeasts were spotted on SD-Leu-Trp (DDO) or SD-Leu-Trp-His (TDO) medium with 2.5 mM 3-Amino-1,2,4-triazole (3-AT) in 10-, 100-, and 1,000-fold dilutions. The empty vectors were used as controls. F, Interactions of the URL1 and URL1mEAR with N-terminus of TPL2 as shown by LCI assay in the same *N. benthamiana* leaf. G, Schematic representation of the EAR-URL1 and VP16-URL1 plant expression constructs. H and I, Phenotypes of the EAR-URL1 and VP16-URL1 transgenic plants in the background of the *Url1* mutant. Bars = 30 cm in (H) and 1cm in (I). J, Cross section of leaf in the EAR-URL1 and VP16-URL1 transgenic plants. Bar = 0.5 cm.

The ethylene-responsive element binding factor-associated amphiphilic repression (EAR) motif is a transcriptional repression domain. Proteins containing this motif negatively regulate the expression of diverse genes (Kagale et al., 2010). A typical EAR motif (LDLDL) is located at the N-terminus of URL1 protein (Figures 2, C and 5, A), suggesting that URL1 may also function as a transcriptional repressor. Therefore, we measured its transcriptional activity using the luciferase transient expression assay in rice protoplasts. In the reporter construct, the *LUC* gene was driven by the CaMV35S promoter fused with five copies of GAL4-binding site (Aguilera et al., 2007). In the effector constructs, URL1 was fused in-frame with the GAL4 DNA-binding domain (GAL4DBD) or GAL4DBD-VP16 (Sadowski et al., 1988). To confirm the effect of the EAR motif on transcriptional activity, the last two conserved Leu residues were replaced by Ser residues (Figure 5B). Each of the six effector constructs with the reporter construct (Figure 5C) was co-transformed into rice protoplasts. As expected, LUC activity was strong in those protoplasts transformed with GAL4DBD or GAL4DBD-VP16. However, LUC activity was severely attenuated in protoplasts transformed with GAL4DBD-URL1 or GAL4DBD-URL1-VP16 (Figure 5D), indicating that URL1 functions as a transcriptional repressor. Mutation in the EAR motif (URL1mEAR) partially relieved this repression (Figure 5D).

The EAR motif can recruit the TPL family proteins, forming a transcriptional repression complex (Szemenyei et al., 2008; Pauwels et al., 2010). Thus, we examined potential interactions between URL1 and TPL2, the rice homolog of TPL, using yeast two-hybrid and firefly luciferase complementation imaging (LCI) assays. URL1 could interact with the N-terminal region of TPL2 *in vitro* and *in vivo* (Figure 5, E and F), which is known to be sufficient for mediating the interaction between TPL and EAR-containing proteins (Causier et al., 2012; Zhang et al., 2017). Mutation in the EAR motif (URL1mEAR) weakened the interaction between URL1 and TPL2 (Figure 5, E and F), suggesting that the interaction between URL1 and TPL2 depends on the EAR motif.

To ascertain the role of URL1 transcriptional repression activity in the regulation of leaf rolling, four tandem copies of the exogenous EAR motif were fused to the N-terminus of *URL1* cDNA and expressed under the maize *ubiquitin1* promoter (Figure 5G). The construct was transformed into the *Url1* mutant. The transgenic lines displayed extremely adaxially rolled leaves (Figure 5, H and I). Observation of leaf cross-sections showed severe leaf rolling with one edge of leaf blade completely enwrapped by the other (Figure 5J). Besides, the *EAR-URL1* transgenic plants were dwarf and sterile (Figure 5H). In contrast, fusion of the VP16 activation domain to *URL1* had no effect on leaf rolling and plant height.

**Figure 6:**
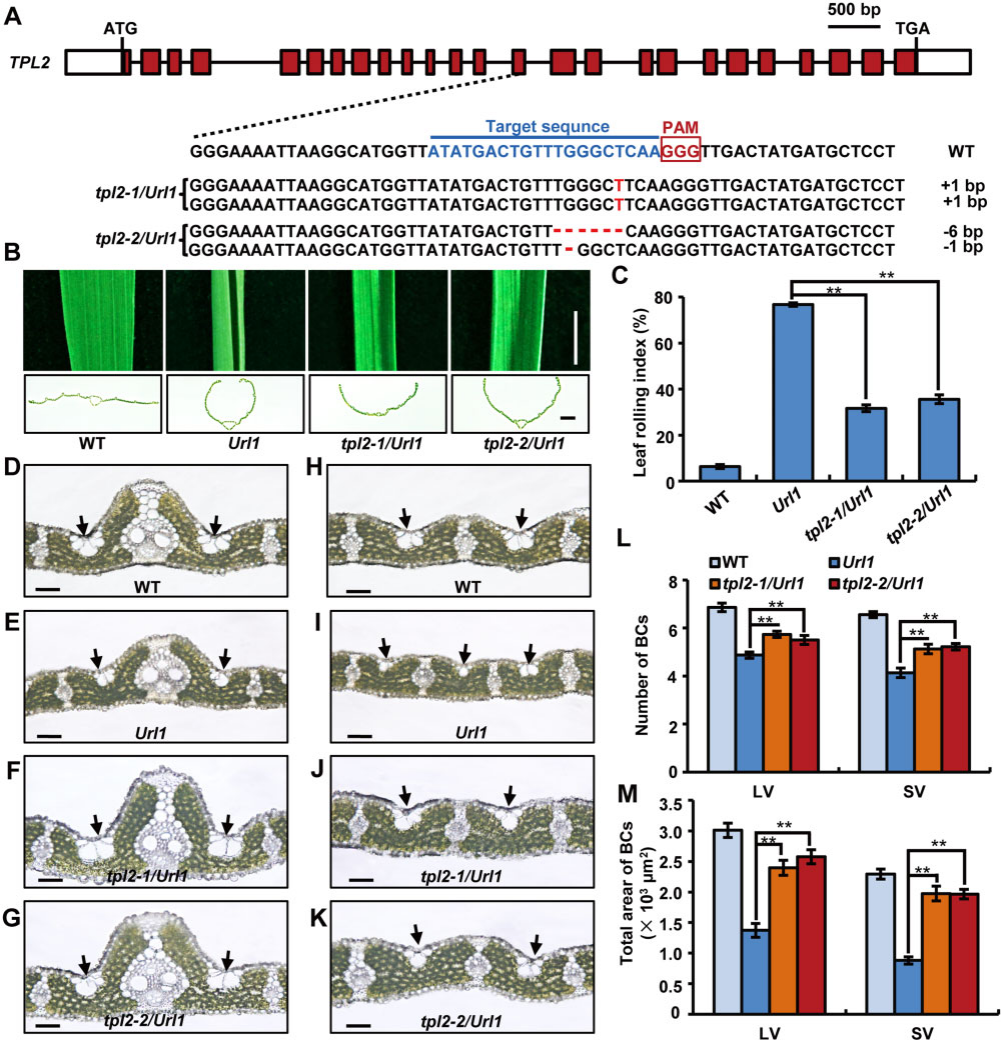
TPL2 is required for URL1 to regulate adaxial rolling of leaf in the *Url1* mutant. A, The gene structure of *TPL2* and the mutated sequence of *tpl2/Url1* mutants generated via CRISPR-Cas9. The homozygous *tpl2-1/Url1* mutant contains a 1-bp insertion (red letter). The biallelic *tpl2-2/Url1* mutant harbors 6-bp and 1-bp deletions (dashed lines). Exons and introns are represented by red boxes and black lines, respectively. The target sequence is indicated in blue and the PAM is highlighted with a red box. B, Leaf blade (upper panel) and cross section (lower panel) of WT, *Url1* and *tpl2/Url1*. Bars = 1 cm in (upper) and 1 mm in (lower). C, LRI of WT, *Url1* and *tpl2/Url1*. Data are presented as mean *±* se (*n *=* *15). Significance of data is tested by Student’s *t* test (**P <* *0.01). D–K, Transverse section of the mature leaf blade of *tpl2/Url1* mutants to show the morphological characteristics of bulliform cells near large veins (D–G) and small veins (H–K). The black arrows indicate bulliform cells. Bars = 50 *μ*m. L and M, Bulliform cell numbers (L) and area (M) of WT, *Url1* and *tpl2/Url1*. Data are presented as mean *±* se (*n *=* *15). Significance of data is tested by Student’s *t* test (**P <* *0.01).

To clarify the relationship between URL1 and TPL2 in regulating leaf rolling, we created the *TPL2* knockout mutants in the *Url1* mutant background using CRISPR/Cas9 technology, and characterized two independent mutant alleles, *tpl2-1/Url1* and *tpl2-2/Url1* (Figure 6A). The adaxially rolled leaf phenotype of *Url1* was partially alleviated in the *tpl2-1/Url1* and *tpl2-2/Url1* mutants (Figure 6B). The LRI values of *tpl2-1/Url1* and *tpl2-2/Url1* were 32% and 36%, respectively, which were greatly reduced compared with that of the *Url1* mutant (77%; Figure 6C). Further microscopic observations revealed that *tpl2-1/Url1* and *tpl2-2/Url1* had more and larger bulliform cells than *Url1* (Figure 6, D–M). Therefore, the transcriptional corepressor activity of TPL2 is required for URL1-regulated adaxial leaf rolling in the gain-of-function *Url1* mutant.

### URL1 interacts physically with ROC5

**Figure 7:**
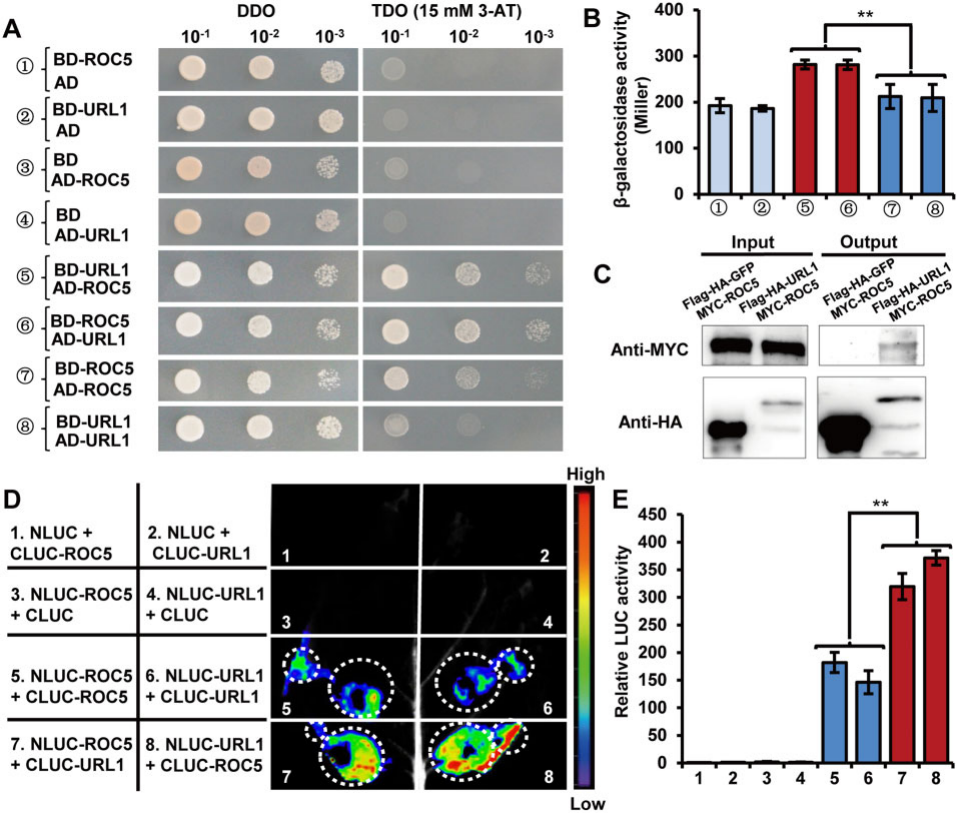
URL1 interacts directly with ROC5 protein. A, URL1 interacted with ROC5 in the yeast two-hybrid assay. Transformed yeasts were spotted on SD–Leu–Trp (DDO) or SD–Leu–Trp–His (TDO) medium with 15 mM 3-AT in 10-, 100-, and 1,000-fold dilutions. The empty vectors were used as controls. B, The liquid β-Galactosidase activity assay in yeast clones containing different combinations of bait and prey. Data are presented as mean ± se (*n *=* *3). Significance of data is tested by Student’s *t* test (**P <* *0.01). C, Co-IP analysis. The MYC–ROC5 fusion protein was transiently expressed in the protoplasts of transgenic rice plants B320 (harboring *ProActin1-Flag:HA:Url1*) and B6 (harboring *ProActin1-Flag:HA:GFP*; negative control), respectively. D, The protein–protein interactions of URL1 and ROC5 were analyzed by LCI assay in the same *N. benthamiana* leaf. E, Quantification of LUC activities in the leaves shown in (D). Data are presented as mean ± se (*n *=* *4). Significance of data is tested by Student’s *t* test (**P <* *0.01).

ROC5, another member of the HD-ZIP IV family in rice, modulates leaf rolling via regulation of bulliform cell development (Zou et al., 2011). This prompted us to check whether URL1 and ROC5 interact with each other. Both yeast two-hybrid and co-immunoprecipitation (Co-IP) assays showed that URL1 was able to interact with ROC5 *in vitro* and *in vivo*, respectively (Figure 7, A and C). It was also observed that URL1 and ROC5 themselves could form a homodimer in yeast (Figure 7A). The quantitation of β-galactosidase activity revealed that the strength of the URL1–ROC5 interaction was higher than the URL1–URL1 and ROC5–ROC5 interactions (Figure 7B). To further compare the interaction strength among the URL1–URL1, ROC5–ROC5, and URL1–ROC5 combinations *in vivo*, we performed LUC complementation imaging assays in *Nicotiana benthamiana* leaves (Figure 7D). Strong fluorescence was detected for the four coexpression combinations of NLUC-ROC5 + CLUC-ROC5, NLUC-URL1 + CLUC-URL1, NLUC-ROC5 + CLUC-URL1 and NLUC-URL1 + CLUC-ROC5 (Figure 7D). Quantitative analysis showed that the relative LUC activities were significantly higher in coexpression combinations of NLUC-ROC5 + CLUC-URL1 and NLUC-URL1 + CLUC-ROC5 than those in coexpression combinations of NLUC-ROC5 + CLUC-ROC5 and NLUC-URL1 + CLUC-URL1 (Figure 7E), suggesting that the URL1 and ROC5 heterodimer is more easily formed than the ROC5/ROC5 or URL1/URL1 homodimers *in vivo*.

**Figure 8:**
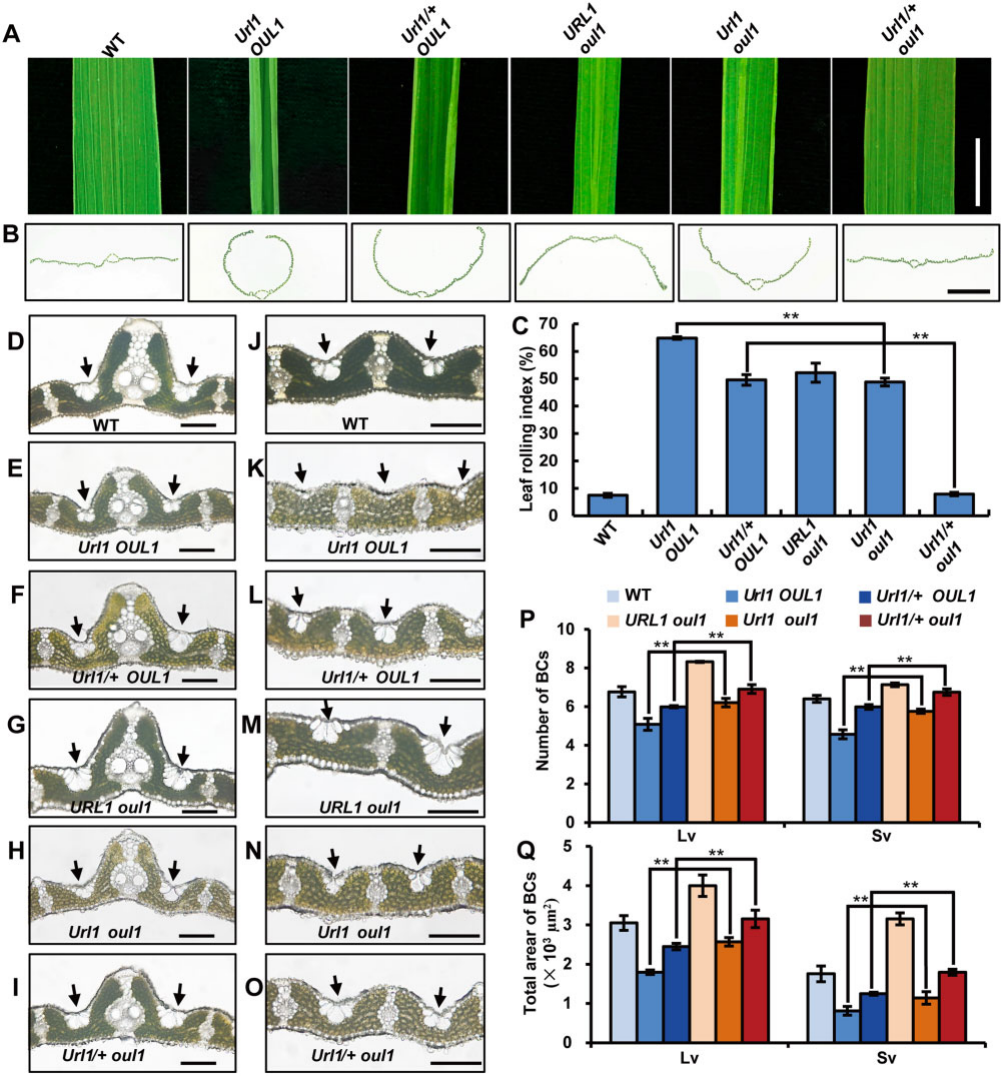
Genetic analysis of *URL1* and *OUL1.* A and B, Mature leaf blade (A) and cross section (B) of WT, *Url1 OUL1*, *Url1/+ OUL1*, *URL1 oul1*, *Url1 oul1*, and *Url1/+ oul1* genotypes. C, LRI of WT, *Url1 OUL1*, *Url1/+ OUL1*, *URL1 oul1*, *Url1 oul1*, and *Url1/+ oul1* plants. Data are presented as mean ± se (*n *=* *15). Significance of data is tested by Student’s *t* test (**P <* *0.01). D–O, Transverse section of the mature leaves from the plants described in (A) to show the morphological characteristics of bulliform cells near large veins (D–I) and small veins (J–O). The black arrows points bulliform cells. Bars = 50 *μ*m. P and Q, Bulliform cell numbers (P) and area (Q) of the plants described in (A). Data are presented as mean *±* se (*n *=* *15). Significance of data is tested by Student’s *t* test (**P <* *0.01).

To clarify their relationship at the genetic level, we crossed the *Url1* mutant with *oul1*, the T-DNA knockout mutant of *ROC5* in Nipponbare background which shows abaxially rolled leaves (Zou et al., 2011). Knockout of *ROC5* in the homozygous *Url1* mutant (i.e. the *Url1 oul1* double mutant) significantly reduced the degree of adaxial leaf rolling compared with the *Url1* single mutant (Figure 8, A–C, the second and fifth sample). Interestingly, knockout of *ROC5* in the heterozygous *Url1* mutant (i.e. the *Url1/+ oul1* mutant) completely suppressed the adaxially semi-rolled leaf phenotype and the leaf became as flat as WT (Figure 8, A–C, the third and sixth sample). Further microscopic observations revealed that both the number and size of bulliform cells increased modestly in the *oul1 Url1* double mutants compared with that in the *Url1* single mutant, and were restored to the WT level in the *Url1/+ oul1* mutant (Figure 8, D–Q). To further confirm their genetic interaction, we knocked out *ROC5* in the *Url1* mutant using CRISPR/Cas9 technology and obtained two homozygous T_0_ mutants with a 1- and 2-bp deletion, respectively. The degree of adaxial leaf rolling was significantly reduced in both lines (Supplemental Figure S8). These results suggest that ROC5 is required for the function of URL1 and that these two proteins probably function as a heterodimer that modulates leaf rolling. Meanwhile, in the absence of ROC5, URL1 may function as a homodimer that regulates leaf rolling.

### *ACL1* is a putative target gene repressed by URL1

**Figure 9:**
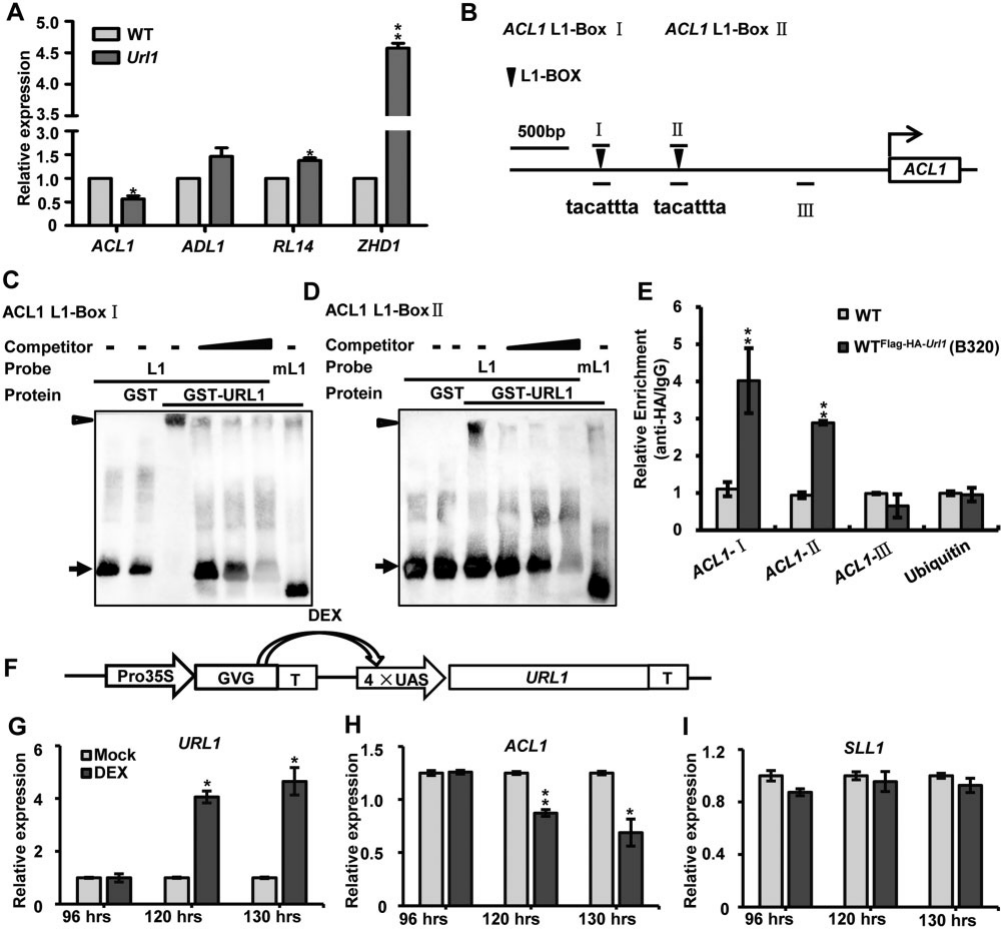
URL1 directly binds to the L1 box in the *ACL1* promoter and inhibits its expression. A, RT-qPCR analysis of expression level of genes related to leaf rolling in *Url1*. Data are presented as mean ± se (*n = *3)*.* Significance of data is tested by Student’s *t* test (*P <* *0.05, **P <* *0.01). B, The positions of L1 box-like sequences in the *ACL1* promoter. C and D, Gel retardation assay were performed using a labeled L1 box probe (L1) derived from the *ACL1* promoter, together with the GST alone or the GST–URL1 fusion protein as indicated. The wedge indicates unlabeled L1 probe DNA in competition assays with increasing amounts of 100-, 300-, and 1,000-fold molar excesses. Assay using mutated probes (mL1) with GST–URL1 is also shown. The black triangles (upper) and arrows (bottom) represent the L1 box binding to URL1 protein and free probes, respectively. E, ChIP assay showing that URL1 binds to the promoter of *ACL1* in vivo. Regions I, II, III detected by ChIP were marked by short lines in (B)*.* An upstream DNA region of *ubiquitin* and *ACL1* without L1 box was used as a negative control. Data are presented as mean ± se (*n *=* *3)*.* Significance of data is tested by Student’s *t* test (**P <* *0.01). F, Schematic representation of the DEX-inducible expression vector. GVG, GAL4 binding domain-VP16 activation domain-glucocorticoid receptor domain; UAS, GAL4 upstream activating sequences. After treatment by DEX, the transcription activator GVG will move from cytoplasm into nucleus and activate downstream gene expression. G–I, RT-qPCR analysis of *URL1* (G) and *ACL1* (H) expression in calli harboring the DEX-inducible expression vector treated with 30 µM DEX or Mock for different hours. The *SLL1* gene was used as a negative control (I). Data are presented as mean ± se (*n *=* *3). Significance of data is tested by Student’s *t* test (*P <* *0.05, **P <* *0.01).

Next, we aimed to identify the downstream target gene regulated by URL1. We analyzed the transcript levels of *ZHD1*, *ADL1*, *ACL1*, and *RL14*, which have been shown to regulate rice leaf rolling by modulating bulliform cell development (Hibara et al., 2009; Li et al., 2010; Fang et al., 2012; Xu et al., 2014). *ACL1* was significantly downregulated, whereas *ZHD1*, *ADL1*, and *RL14* were upregulated in the *Url1* mutant (Figure 9A). Considering that URL1 functions as a transcriptional repressor and that *Url1* is a gain-of-function mutant, *ACL1* could be the potential target of URL1.

The L1 box (5′-TAAATGYA-3′) is a well-conserved *cis*-regulatory element in the promoter of target genes regulated by HD-ZIP IV transcription factors (Abe et al., 2003). Interestingly, two L1 boxes were identified at 1,846–1,853 and 2,600–2,607-bp upstream of the start codon of *ACL1* (Figure 9B). Electrophoretic mobility shift assay (EMSA) revealed that glutathione-S-transferase (GST)-URL1 could remarkably reduce the electrophoretic mobility of the probes containing two *ACL1* L1 boxes (Figure 9, C and D), but the GST protein alone could not cause mobility reduction. The binding specificity of URL1 to the L1 box was confirmed by effective competition using an excess amount of unlabeled probe and the absence of mobility shift of the mutated L1 box probes (Figure 9, C and D). Then chromatin immunoprecipitation (ChIP) was performed with the transgenic line B320-1 (Supplemental Figure S6A). DNA regions I and II of *ACL1* (*ACL1-I*, *ACL1-II*), which contained the L1-box, were significantly enriched in the coimmunoprecipitates from the transgenic line. In contrast, there was no enrichment for the negative control region of *ACL1* (*ACL1-III*) or promoter region of the nontarget gene *ubiquitin* (Figure 9E). Therefore, URL1 could physically bind to the *ACL1* promoter via the L1 box.

To examine whether *ACL1* was transcriptionally repressed in response to *URL1* expression *in planta*, we used a *pINDEX* two-component induction system (Figure 9F; Ouwerkerk et al., 2001). The *URL1* transcript increased dramatically upon dexamethasone (DEX) treatment (Figure 9G). Accordingly, the expression level of *ACL1* decreased distinctly upon the induction of *URL1* expression (Figure 9H). In contrast, the noncandidate gene *SLL1* (Zhang et al., 2009) was not evidently repressed by DEX treatment (Figure 9I). The expression levels of these genes were not obviously affected by the DEX treatment in WT rice plants (Supplemental Figure S9). These results indicate that URL1 can repress *ACL1* gene expression.

**Figure 10:**
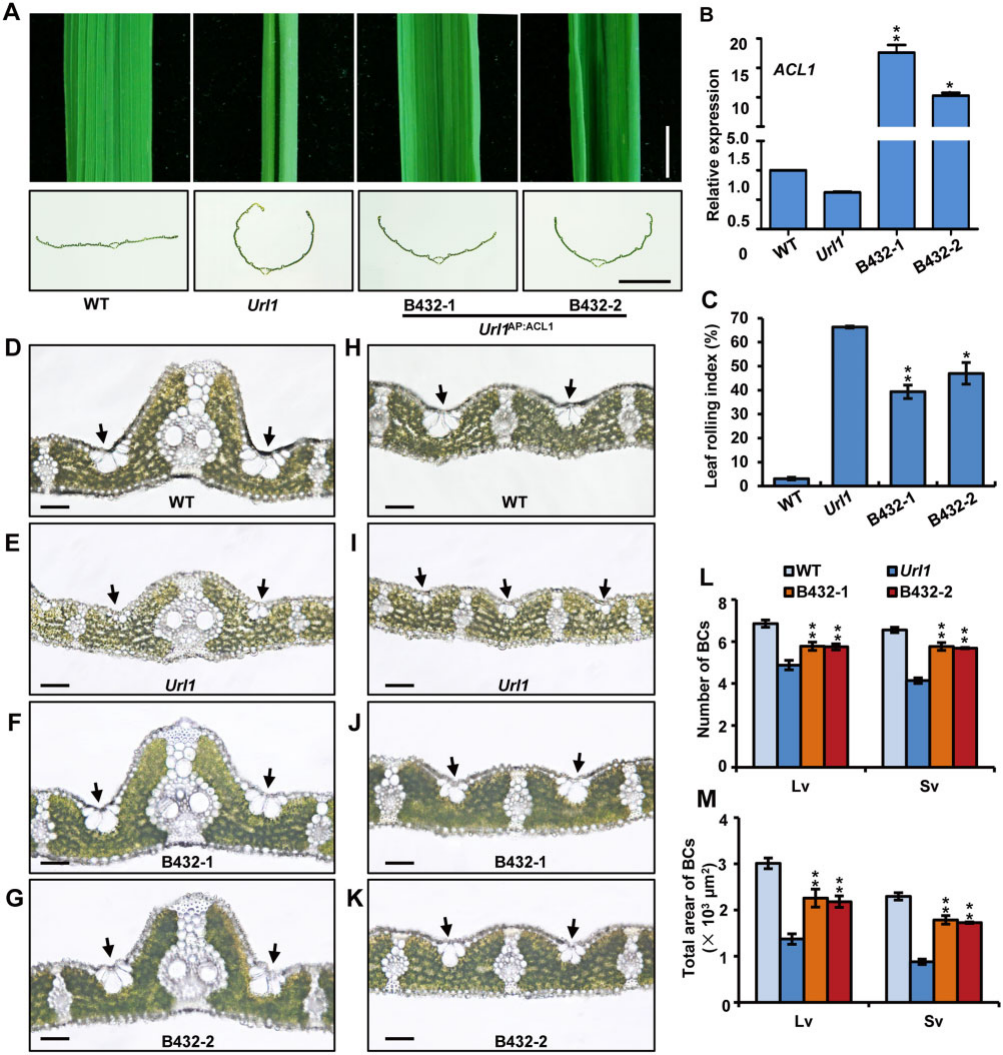
Overexpression of *ACL1* alleviates adaxial rolling of leaf in *Url1*. A, Leaf blade (upper panel) and cross-section (lower panel) of WT, *Url1* and transgenic plants overexpressing *ACL1* in the *Url1* mutant background (B432). Bars = 0.5 cm in (upper) and 0.25 cm in (lower). B, RT-qPCR analysis of *ACL1* expression in the transgenic lines B432. Data are presented as mean ± se (*n *=* *3)*.* Significance of data is tested by Student’s *t* test (**P <* *0.01). C, LRI of WT, *Url1* and transgenic lines B432. Data are presented as mean ± se (*n *=* *15). Significance of data is tested by Student’s *t* test (**P <* *0.01). D–K, Transverse section of the mature leaf blade of B432 to show the morphological characteristics of bulliform cells near large veins (D–G) and small veins (H–K). The black arrows indicate bulliform cells. Bars = 50 μm. L and M, Bulliform cell numbers (L) and area (M) of WT, *Url1* and transgenic lines B432. Data are presented as mean ± se (*n *=* *15). Significance of data is tested by Student’s *t* test (**P <* *0.01).

To examine the functional relationship of ACL1 with URL1 in the modulation of leaf rolling, we overexpressed *ACL1* under control of the rice *Actin1* promoter in the *Url1* mutant (B432). The adaxial rolling of the *Url1* mutant leaf was evidently reduced by the overexpression of *ACL1* (Figure 10A). The decreased LRI was correlated to the increased expression level of *ACL1* in the transgenic lines (Figure 10, B and C). Since *ACL1* regulates leaf rolling by controlling the number and size of bulliform cells (Li et al., 2010), we further made microscopic observation of the leaves in B432 transgenic plants. Both the number and size of bulliform cells was significantly increased in B432 when compared with those in the *Url1* mutant, suggesting that overexpression of *ACL1* could partially rescue the adaxially rolled leaves of *Url1* via modulating bulliform cell development (Figure 10, D–M).

## Discussion

### URL1 negatively regulates bulliform cell formation and development

In this study, we identified URL1 as a transcriptional suppressor to modulate leaf rolling through mutant analysis. In the gain-of-function *Url1* mutant, both the number and size of bulliform cells decreased and the leaf rolled adaxially (Figure 1). In contrast, in the CRISPR/Cas9-mediated *URL1* knockout line, both the number and size of bulliform cells increased and the leaf rolled abaxially (Supplemental Figure S4). Therefore, our data strongly support that *URL1* negatively regulates the formation and development of bulliform cells. However, localization of bulliform cells between two vascular bundles and polarized distribution of bulliform cells along the adaxial–abaxial axis did not change in *Url1* (Figure 1). Consistent with an obvious phenotype at early growth stage, *URL1* is predominantly expressed in young leaves rather than mature leaves (Figure 4; Supplemental Figure S7), indicating that *URL1* is directly involved in the establishment rather than maintenance of bulliform cells in the adaxial epidermis.

Besides its role in bulliform cell development, URL1 may also regulate the development of other epidermal cells. First, *URL1* expression is not specifically limited to the bulliform cells, but is also observed in the entire L1 layer of SAM, axillary meristem, leaf primordium, and young leaves (Figure 4, B–D and H; Supplemental Figure S7, L–N). Second, as evident in the semithin cross-sectional analysis of leaf blades, other epidermal cells besides bulliform cells also became smaller in the gain-of-function *Url1* mutant (Supplemental Figure S1) and larger in the loss-of-function *URL1* knockout line (Supplemental Figure S4, D–I and L), respectively. Therefore, URL1 possibly regulates the size of all cells in the L1 layer of leaves. However, these nonbulliform epidermal cells showed comparable changes in cell size at both the abaxial and adaxial sides of leaf blade (Supplemental Figure S1C), and thus had no effect on leaf rolling. In contrast, bulliform cells are present only on the adaxial side of leaf blade and are highly linked to leaf rolling in rice and other grasses (O'Toole et al., 1979; Kadioglu and Terzi, 2007). Therefore, changes in the number and size of bulliform cells may largely account for the leaf-rolling phenotypes in *Url1* gain-of-function mutant and *URL1* knockout lines.

### Potential roles of the two conserved motifs in 3′-UTR in fine-tuning *HD-ZIP IV* mRNA stability

Previous studies showed that most *HD-ZIP IV* genes in plants contain two evolutionarily conserved motifs in their 3′-UTR (Javelle et al., 2011; Zalewski et al., 2013; Burgess and Freeling, 2014). These two motifs are partially complementary and predicted to form a stem-loop structure via base pairing, which may regulate mRNA stability or translation efficiency (Javelle et al., 2011; Klein-Cosson *et al.*, 2015). The two characteristic motifs of HD-ZIP IV genes are also present in the 3′-UTR of *URL1*. We provide several lines of evidence to support its role in fine-tuning *HD-ZIP IV* mRNA stability. First, we found the potential cleavage sites within or surrounding the two conserved motifs (Figure 3, B and C). Second, the nuclear run-on assay proved that nascent transcript level was not altered in the *Url1* mutant, excluding the possibility that the C679T mutation affects transcription (Figure 3E). Third, the mutated *URL1* (C679T) mRNA degraded slower than the WT *URL1* mRNA after inhibiting transcription using cordycepin, suggesting that the mutated *URL1* mRNA has increased stability (Figure 3F).

Although we have revealed the essential role of the *URL1* 3′-UTR in fine-tuning mRNA stability, the underlying molecular mechanism awaits further study. In mammals, the stem-loop structure in the 3′-UTR has been characterized as an mRNA destabilizing element (Paschoud et al., 2006; Mullen and Marzluff, 2008; Leppek et al., 2013; Chang et al., 2016). Stem-loop–binding proteins (SLBPs) such as SLBP, Roquin, and Roquin2 promote degradation of the histone and tumor necrosis factor-α mRNA after binding to the stem-loop structure (Mullen and Marzluff, 2008; Leppek et al., 2013). Hence, we speculate that the stem-loop structure formed in the 3′-UTR of *URL1* may also serve as a binding site for certain proteins that promote mRNA degradation. The WT *URL1* mRNA level was fine-tuned due to the stem-loop structure formed between the conserved CNS1 and CNS2 motifs in the 3′-UTR. In the *Url1* mutant, the C679T substitution in the CNS2 motif reduced the complementarity between CNS1 and CNS2, making the stem-loop structure unstable. This may explain why WT *URL1* mRNA did not over-accumulate in B197 transgenic rice plants, which did not show the adaxial leaf-rolling phenotype (Supplemental Figure S6, B and C). Our study provides an alternative mechanism for expression regulation of *HD-ZIP IV* family genes besides the translational repression proposed by a previous study on the maize *OCL1* gene (Klein-Cosson et al., 2015).

### URL1 serves as a transcriptional repressor by recruiting corepressor TPL2 via its EAR motif

Most EAR motif-containing proteins negatively regulate the expression of their target genes (Kagale et al., 2010). In agreement, we provided several lines of evidence to prove that URL1 serves as a transcriptional repressor in rice and that the EAR motif is required for recruiting the co-repressor TPL2 (Figure 5). More importantly, the interaction between URL1 and TPL2 is required for the proper function of URL1 in regulating leaf rolling. Knockout of *TPL2* in the *Url1* mutant background significantly reduced adaxial rolling of the *Url1* leaf, partly by increasing the expression of *ACL1*, the target gene of *URL1* (Figure 6; Supplemental Figure S10J). However, the leaf-rolling phenotype was not completely suppressed in the *tpl2*/*Url1* mutants (Figure 6, B and C), although the expression level of *ACL1* in the *tpl2*/*Url1* mutants was significantly higher than that in WT (Supplemental Figure S10J), suggesting that besides *ACL1*, TPL2 might target other genes in the modulation of leaf rolling. TPL and TPL-RELATED (TPR) proteins generally interact with different transcription factors and mediate transcriptional repression in a variety of development processes (Liu and Karmarkar, 2008; Szemenyei et al., 2008; Pauwels et al., 2010; Zhu et al., 2010; Causier et al., 2012; Wang et al., 2013; Hao et al., 2014). Consistently, the *TPL2* knockout plants in the *Url1* mutant background exhibited pleiotropic phenotypes. Spikelets on the upper part of the panicle showed enlarged sterile lemma (Supplemental Figure S10, C and D) and reduced stamen number (Supplemental Figure S10, G–I). Spikelets on the lower part of the panicle were pale white and degenerated (Supplemental Figure S10C).

In Arabidopsis, several HD-ZIP IV family members such as ATML1, PDF2, and GL2 directly and negatively regulate the expression of target genes (San-Bento et al., 2014; Lin et al., 2015). PDF2, GL2, and five other members of the Arabidopsis HD-ZIP IV family were predicted to harbor an EAR motif (Kagale et al., 2010). However, transcriptional repression activity has not been experimentally verified for any of them. Our work establishes transcriptional repression activity for HD-ZIP IV family members, which are supposed to act as transcriptional activators (Depege-Fargeix et al., 2011; Peterson et al., 2013).

### URL1 cooperates with ROC5 in modulating leaf rolling

Interestingly, URL1 interacts with ROC5 in yeast and *in planta* (Figure 7); both of these proteins negatively regulate bulliform cell development. Loss-of-function mutation of either *ROC5* (Zou et al., 2011) or *URL1* (Supplemental Figure S4) resulted in increased number and size of bulliform cells and abaxially rolled leaves, suggesting that they are not functionally equivalent. The adaxially rolled-leaf phenotype resulting from the gain-of-function mutation of *URL1* was alleviated when *ROC5* was knocked out (Figure 8; Supplemental Figure S8). In addition, instead of the semi-rolled leaf of the *Url1*/+ heterozygote (Figure 8), the *Url1/+ oul1* mutant showed a flat-leaf phenotype comparable to WT, suggesting that *ROC5* is required for the proper function of URL1. However, the *Url1 oul1* double mutant still exhibited adaxially semi-rolled leaves. These genetic data suggest that *ROC5* is required but not essential for *URL1* function in modulating leaf rolling. Considering that the URL1/ROC5 heterodimer exhibited higher interaction strength than the URL1/URL1 or ROC5/ROC5 homodimers (Figure 7, A, B, D, and E), we speculate that URL1 and ROC5 preferentially function as a heterodimer in modulating leaf rolling. Meanwhile, URL1 can form a homodimer in the *Url1* background, which accumulated more *URL1* transcripts than WT, and regulate leaf rolling.

It is noteworthy that ROC5 also has a canonical EAR motif at the N-terminus (Figure 5A). Both yeast two-hybrid assay and luciferase complementation assay in *N. benthamiana* leaves confirmed that ROC5 can also interact with TPL2 *in vitro* and *in vivo* (Supplemental Figure S11). Therefore, URL1, ROC5, and TPL2 may form a transcriptional repression complex. HD-Zip IV family proteins have been shown to recognize the L1 box motif (5′-TAAATGT-3′; Ohashi et al., 2003; Nakamura et al., 2006). Our data show that URL1 can bind with the L1 boxes in the promoter of the *ACL1* gene and suppress its expression (Figure 9), thus negatively regulating a positive regulator of bulliform cell development.

**Figure 11:**
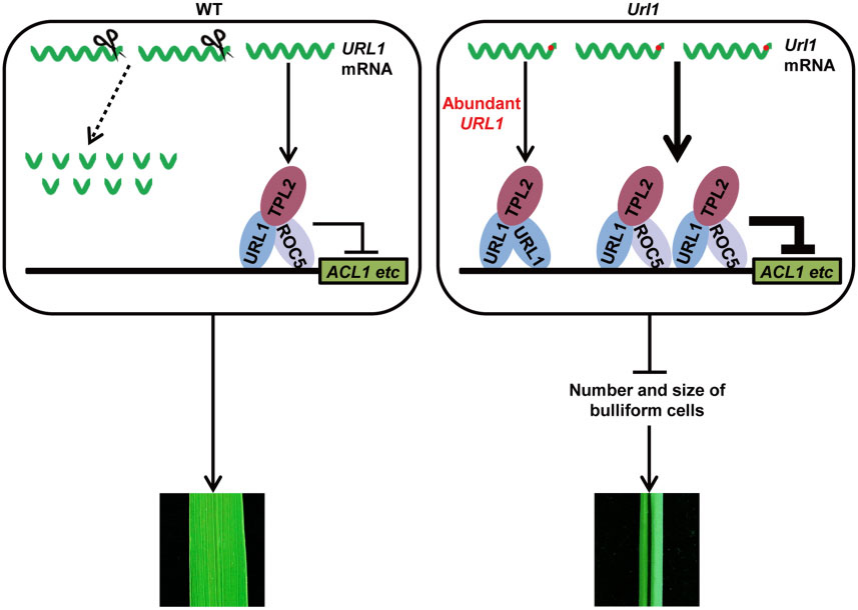
A working model for the *URL1* gene in the regulation of adaxial rolling of leaf. In WT, a part of the *URL1* transcripts were cleaved and degaraded due to the stem-loop structure mediated by the conserved CNS1 and CNS2 motifs in the 3′-UTR. The URL1 protein produced from the remaining transcript and ROC5 form a heterodimer and interact with the transcriptional corepressor TPL2 through the EAR motif to inhibit the expression of URL1 target genes such as *ACL1* that regulates bulliform cell development, maintaining flat leaves. In the *Url1* mutant, a C-to-T substitution in the second motif CNS2 of 3′-UTR increased the mRNA stability and abundance. The abundant URL1 protein would form a homodimer as well as the URL1/ROC5 heterodimer to further repress the URL1 target genes, leading to a reduction in the number and size of bulliform cells to result in adaxially rolled leaves.

Collectively, our study integrates several genes controlling bulliform cell development into a regulatory network as shown in Figure 11. A transcriptional repression complex composed of URL1, ROC5, and the transcriptional corepressor TPL2 suppresses expression of the *ACL1* gene and modulates leaf rolling consequently. In WT, the stem-loop structure mediated by the conserved CNS1 and CNS2 motifs in the 3′-UTR triggers degradation of a portion of the *URL1* mRNA. The modest amount of URL1 protein produced from the remaining *URL1* transcript interacts with ROC5 and recruits corepressor protein TPL2, which inhibits the expression of target genes and maintains flat leaves. In the *Url1* mutant, a C-to-T substitution in the second motif CNS2 of 3′-UTR increased the *URL1* mRNA stability and abundance. The excessive amount of URL1 protein would form a homodimer in addition to the URL1/ROC5 heterodimer, thus further repressing URL1 target genes, leading to a reduction in the number and size of bulliform cells and eventually adaxially rolled leaves. In addition to *ACL1*, URL1 may also regulate other target genes. Genome-wide identification of URL1 target genes will further elucidate the role of URL1 in bulliform cell development.

## Materials and methods

### Plant material and growth conditions

Rice plants were grown in the paddy fields of the Chinese Academy of Agricultural Sciences (Beijing, China) under natural growing conditions. Seedlings for protoplast isolation, ChIP, Co-IP, etc. were grown in a phytotron with 16-h light (30°C) and 8-h dark (22°C). The LRI of flag leaves at maturity was calculated by the following formula: LRI (%) = (Lw−Ln)/Lw*100. Ln and Lw indicated the distance of leaf blade margins at natural state and the distance of leaf blade margins at unfolding state, respectively (Shi et al., 2007).

### Histology and microscopy observations

For paraffin sectional analysis, the middle part of each leaf was taken at the time of anthesis and fixed in FAA solution followed by dehydration using a graded ethanol series. Sections 5- to 6-*μ*m thick were cut and photographed as described previously (Fang et al., 2018). The longitudinal arrangement of bulliform cells were analyzed using mature leaf blade (12th leaf) as described previously (Zou et al., 2011). For semithin sectional analysis, the mature leaves were cut into small pieces and fixed in 2.5% (v/v) glutaraldehyde overnight at room temperature. Samples were subsequently washed three times in phosphate buffer (pH 7.2), dehydrated in ethanol, and embedded in Spurr resin as described previously (Zhao et al., 2002). The embedded samples were cut into 1-μm sections using an RMC MT-7 ultramicrotome (Reichert-Jung, Depew, NY, USA). Semithin sections were stained with 0.25% (w/v) TBO and photographed under an Olympus BX53 microscope. The program ImageJ (http://rsb.info.nih.gov/ij/) was used to measure the epidermal cell area at the abaxial side of the leaf.

### Map-based cloning of *URL1*

To map the *URL1* locus, 309 plants showing an extremely rolled leaf phenotype in the F_2_ population derived from a cross between *Url1* and Dular and newly developed InDel markers were used (Supplemental Table S1). To identify the mutation site, genomic DNA fragments of candidate genes were amplified from the *Url1* mutant and the PCR products were sequenced directly. 3′-UTR sequence of *URL1* was determined using a Smart RACE cDNA amplification kit (Clontech-Takara Bio).

### Constructs for transgenic plants

To construct the genomic DNA complementation vector, the 5,956-bp *URL1* genomic DNA sequence (including 1,940-bp upstream of ATG, and 946-bp downstream of TGA) was amplified from the *Url1* mutant and cloned into binary vector pCAMBIA1305.1. To construct the *URL1* gene overexpression vector, the *URL1* coding sequence (CDS) plus the 3′-UTR was amplified from the WT and *Url1* mutant leaf cDNA and ligated into the binary vector pCAMBIA1305.1-APFHN (Supplemental Figure S12). To construct the *URL1* RNA interference vector, a 174-bp cDNA fragment of *URL1* was ligated in the LH-FAD2-1390RNAi vector (Li et al., 2013) in opposite orientation. To construct the *URL1*, *TPL2*, and *ROC5* CRISPR-Cas9 vectors, the target sequences of sgRNAs were selected using the web-based tool CRISPR-P (Lei et al., 2014). The OsU6b-URL1T1, OsU3-TPL2T1, and OsU3-ROC5T1 sgRNA expression cassettes were assembled in the plant binary vector pYLCRISPR/Cas9 Pubi-H, as described previously (Ma et al., 2015). To construct the maize *ubiquitin1* promoter-driven EAR:URL1 and VP16:URL1 expression vectors, PCR primers attB1-URL1F/R were designed to amplify the URL1 CDS plus 3′-UTR from the wild-type cDNA. BP reaction with URL1 attB-flanked fragment and pDONR/Zeo vector was performed to generate the *URL1* entry clone. We then performed LR reaction with *URL1* entry clone and the plant binary vector LP041 nEAR-hyg-asRED or LP042 nVP64-hyg-asRED (Zhao et al., 2015). To construct the DEX-inducible expression vector of *URL1*, the *URL1* CDS plus 3′-UTR was ligated into vector pINDEX3 (Ouwerkerk et al., 2001). To construct the *URL1* promoter-driven *GUS* reporter gene, a 2,566-bp fragment upstream of the *URL1* start codon was inserted in pCAMBIA1305.1. To construct the rice *actin1* promoter driven *ACL1* over-expression vector, the CDS plus the 5′- and 3′-UTR of *ACL1* gene was ligated into pCAMBIA1305.1-APFHC (Supplemental Figure S12). All the primers used in vector construction are listed in Supplemental Table S2.

### Modified RNA ligase-mediated 5′-RACE

Modified RLM 5′-RACE was performed using the FirstChoice RLM-RACE kit (Invitrogen). Poly A+ mRNA was purified from 1 mg total RNA extracted from 2-week-old seedlings using Oligotex (Qiagen). The purified mRNA (without calf intestine alkaline phosphatase treatment) was ligated to 5′-RACE RNA adaptors for reverse transcription. The cDNA samples were amplified by nested PCR using the gene-specific primers URL1(UTR)R1 (5′-TTTTTTCCATTTATAACCGAAATG-3′) and URL1(UTR)R2 (5′-CCATTTATAACCGAAATGAATGTTC-3′) for the first and second round PCR, respectively. The second round PCR products were cloned in *pEASY*-Blunt Simple Cloning Vectors (Transgen Biotech) and sequenced.

### Nuclear run-on assay

Nuclear run-on assay was performed as described previously with slight modifications (Roberts et al., 2015). Briefly, 6 g of 7-d-old *Url1* mutant and WT seedlings were harvested for isolating nuclei by centrifuging using Percoll density gradients (Folta and Kaufman, 2006). The nuclei layer was gently resuspended in 300-*μ*L storage buffer (50 mM Tris–HCl pH 7.8, 10 mM 2-mercaptoethanol, 20% (v/v) glycerol, 5 mM MgCl_2_ and 0.44 M sucrose), snap-frozen in liquid nitrogen, and then stored at –80°C until use. Run-on reaction was based on the incorporation of biotin-16-uridine-5′-triphosphate (biotin-16-UTP) into nascent transcripts (Patrone et al., 2000). Approximately 10^6^ nuclei in 50-*µ*L storage buffer were incubated with 20 U RNAsin (Promega) for 10 min at 30°C to be pre-warmed. Then 10 µL 10-fold pre-warmed transcription buffer (working concentration: 25 mM Tris–HCl pH 7.8, 37.5 mM NH_4_Cl, 5 mM MgCl_2_, 5% v/v glycerol, 1 mM each of ATP, GTP, and CTP) and 1 mM biotin-16-UTP (Roche Diagnostics) was added to each sample and the transcription reaction continued for 45 min at 30°C. The reaction was stopped by placing the samples on ice. Immediately, total RNA was isolated with TRIzol reagent (Invitrogen) and treated with RNase free DNase I (Roche). Total RNA was then incubated with streptavidin magnetic beads (NEB) to immunoprecipitate biotinylated RNAs and used for reverse-transcription with random decamers to generate cDNA (Takara). Nascent *URL1* mRNA levels were measured via RT-qPCR with primers listed in Supplemental Table S3 and the rice *Actin* gene was used as an internal control.

### mRNA decay assay

mRNA decay assay was performed as described previously (Niu et al., 2019). A 7-d-old *Url1* mutant and WT seedlings were immersed in incubation buffer (1 mM PIPES (1,4-Piperazinediethanesulfonic acid), pH 6.25, 1 mM trisodium citrate, 1 mM KCl, 15 mM sucrose) for 30 min. Cordycepin (Sigma C-3394) was supplemented to a final concentration of 200 *µ*g mL^−1^ and samples were vacuum infiltrated for 5 min. Shoot bases were collected at different time points to extract RNA. RT-qPCR analysis was conducted using *ubiquitin* as an internal control. Five biological replicates were used at each time point.

### Reverse transcription quantitative PCR

Total RNA was extracted from different tissues or protoplasts using TRIzol reagent (Invitrogen). First-strand cDNA was synthesized from 2 *μ*g of total RNA using a SuperScript II kit (TaKaRa). RT-qPCR was performed using the ABI prism 7500 real-time PCR System with SYBR Premix Ex Taq kit (TaKaRa). The rice *ubiquitin* gene was used as an internal control. At least three biological replicates were analyzed. The relative gene expression level was calculated using the 2^–ΔΔCT^ method (Livak and Schmittgen, 2001). Primers are listed in Supplemental Table S3.

### Histochemical GUS assay

Histochemical assay of the transgenic rice plants harboring the *URL1pro-GUS* construct was performed as described previously (Jefferson et al., 1987).

### *In situ* hybridization

Shoot bases of 7-d-old seedlings from WT and the *Url1* mutant were fixed in 4% (w/v) paraformaldehyde at 4°C overnight, followed by a dehydration series and infiltration, and embedded in paraffin (Paraplast Plus, Sigma). The tissues were sliced into about 8-*µ*m sections with a microtome (Leica RM2145), and the sections were mounted on RNase-free glass slides. A 292-bp fragment within the 3′-UTR region (Supplemental Table S3) was used as the template to generate sense and antisense RNA probes that were labeled using a DIG Northern Starter Kit (catalog no. 2039672; Roche). RNA *in situ* hybridization with probes was performed on sections.

### Subcellular localization

The *URL1* CDS was ligated in the transient expression vector *pAN580* having the GFP encoding gene and transformed into rice protoplasts (Sheng et al., 2014). GFP signals were observed using a confocal laser scanning microscope at 488 nm (Leica SP2).

### Luciferase transient expression assays in rice protoplasts

The constructs used to examine effect of the *URL1* 3′-UTR on gene expression were prepared as follows. First, the empty reporter construct *pAN121* was developed from the transient expression vector *pAN580* (Sheng et al., 2014). The *LUC*-coding sequence was amplified from the plasmid *Pro35S-GAL4UAS-LUC* (Lin et al., 2013) and ligated into *pAN580* replacing the *GFP* gene. Then, the *URL1* 3′-UTR was amplified from the WT and *Url1* mutant genomic DNA and inserted in the *pAN121* vector. The internal control vector *p35S-RLUC* was developed from *pAN580* by replacing the *GFP* gene with the *Renilla LUC* gene. The fusion constructs used to examine the transcriptional repression activity of the URL1 protein were made as follows. The reporter plasmid *Pro35S-GAL4UAS-LUC* was adopted from a previous study (Lin et al., 2013). The empty effector plasmids *Pro35S-GAL4BD* and *Pro35S-GAL4BD:VP16* were constructed previously (Peng et al., 2017). To create the effector construct without the VP16 motif, the coding sequences of *URL1* was inserted into *Pro35S-GAL4BD:VP16* vector using primers URL1BBF/R. To create the effector construct containing the VP16 motif, the coding sequences of *URL1* was ligated into *Pro35S-GAL4BD:VP16* vector using primers URL1BKF/R. Protoplasts were isolated from rice seedlings and transformed as described previously (Yoo et al., 2007). In the assay for URL1 transcriptional repression activity, 5 *µ*g of reporter plasmid, 4 *µ*g of effector plasmid, and 1 *µ*g of internal control plasmid *pAN508* (*Pro35S-GUS*) were used for each transformation event. The firefly luciferase activity was measured on a Centro XS³ LB 960 High Sensitivity Microplate Luminometer (Berthold Technologies). The GUS activity of the same sample was measured using the GUS extraction buffer on fluorescence spectrophotometer F-4600 (Hitachi). The relative activity of the different reporter constructs is expressed as the LUC/GUS ratio. Three biological replicates were used. In the assay for the *URL1* 3′-UTR regulatory roles on gene expression, 5 *µ*g of reporter plasmid and 1 *µ*g of internal control plasmid (*p35S-Renilla LUC*) was added to each transformation. The fireﬂy and *Renilla* luciferase activities were monitored using the Dual-Luciferase Reporter Assay System (Promega). The relative activity of different reporter constructs was calculated as the LUC/RLUC ratio.

### Yeast two-hybrid analysis

N-terminus of *TPL2* was cloned into bait vector *pGBKT7*. Full-length coding sequence of *URL1, URL1mEAR*, and *ROC5* were respectively cloned into prey vector *pGADT7*. To test the dimerization between URL1 and ROC5, the full-length CDS of *URL1* and *ROC5* were cloned into *pGBKT7* or *pGADT7*. Different combinations of prey and bait vectors were co-transformed into yeast stain AH109 as described previously (Zhang et al., 2017).

### Luciferase complementation imaging assay

The full-length *URL1*, *URL1mEA*R, *ROC5*, and N-terminus of *TPL2* were fused with the N- or C-terminal parts of the reporter gene *LUC* to generate *CLUC-URL1*, *CLUC-URL1mEAR*, *CLUC-ROC5*, and *NLUC-TPL2*. All the constructs were transformed into *Agrobacterium tumefaciens* strain EHA105. The bacteria with OD_600_ = 0.5 were collected and resuspended using activity buffer (10 mM Morpholinoethanesulfonic acid (MES), pH 5.7, 10 mM MgCl_2_, 150 mM acetosyringone). An equal volume of *A*. *tumefaciens* harboring different CLUC and NLUC construct pairs were mixed to a final concentration of OD_600_ = 1.0. Four or six different combinations of *A. tumefaciens* were infiltrated into different positions in the same leaf of *N. benthamiana*. The plants were placed in the dark for 12 h followed by 72 h in a growth chamber under normal conditions. The infiltrated leaves were sprayed with 100 mM luciferin and kept in darkness for 10 min. LUC signals were captured with the NightSHADE LB 985 (Berthold Technologies). Quantitative analysis was performed using IndiGo software (Berthold Technologies).

### Co-immunoprecipitation

The *ROC5* CDS (coding sequence) was cloned into *pCM1307-N-MYC* vector. The resulting *Pro35S-6×Myc:ROC5* construct was transformed into the protoplasts of transgenic rice plants B320 (harboring *ProActin1-Flag:HA:Url1*) and B6 (harboring *ProActin1-Flag:HA:GFP*). After incubation overnight, protoplasts were lysed, and the supernatant was incubated with anti-HA-conjugated agarose. The co-immunoprecipitated proteins were detected by immunoblotting with anti-Myc and anti-HA antibodies.

### EMSA and ChIP assays

The *GST–URL1* fusion construct was developed by cloning full-length CDS of *URL1* into *pCold-GST* vector. The fusion protein was expressed in the *Escherichia coli* strain BL21 and purified using Glutathione Sepharose 4B (GE Healthcare). The 70-bp probes encompassing the L1 box in the *ACL1* promoter (Supplemental Table S4) were labeled at the 3′-end with biotin and annealed to form the double-stranded DNA probe before use. As the negative controls, probes with a complete deletion of the *ACL1* L1-box were also prepared. 100-, 300-, and 1,000-fold molar excesses of unlabeled probes were used as competitors in the competing assays. EMSA (Electrophoretic mobility shift assay) was performed using the LightShift Chemiluminescent EMSA Kit (Thermo Scientific Cat. No. 20148). The labeled probes and their shifted protein complexes were detected in an automatic chemiluminescence image analysis system (Tanon 5200).

The ChIP assay was performed as previously described with slight modifications (Gendrel et al., 2005). Briefly, 1 g tissue samples of 14-d-old seedlings of the *URL1* over-expression line B320 and WT were cross-linked with 1% (v/v) formaldehyde. The isolated chromatin complex was sonicated to 0.2- to 1.0-kb fragments, and divided into two aliquots. After keeping 1% of each aliquot as the input DNA, each aliquot was immunoprecipitated with anti-HA antibody or IgG serum and incubated at 4°C overnight. The immunocomplex was collected using Protein A agarose beads (Sigma, P3476). After washing, through elution and reversing the cross-link, the precipitated DNA was recovered. The enrichment of particular regions in the *ACL1* promoter (Supplemental Table S4) was quantified by qPCR as described previously.

### DEX treatment

Calli from WT and the T_1_ seeds of *pINDEX3-URL1* transgenic rice plants were induced. For the DEX or mock treatment, the transgenic calli were transferred to solid MS medium plates containing 30 µM DEX (Sigma, D1756) or equal volumes of DMSO. Samples were harvested at 96, 120, and 130 h after treatment and used for RNA extraction. Three biological replicates were used at each time point for analysis, and it was repeated three times.

### Accession numbers

Sequence data from this article can be found in the GenBank databases or the Rice Genome Annotation Project website (http://rice.plantbiology.msu.edu/) under the following accession numbers: *URL1* genomic DNA, MH822134; *URL1* cDNA, MH822135; *ROC5*, LOC_Os02g45250; *TPL2*, LOC_Os08g06480; *ACL1*, LOC_Os04g33860.

## Supplemental data

The following supplemental materials are available.

**Supplemental Figure S1.** Cross section of the wild type (WT) and *Url1* mutant mature leaf.

**Supplemental Figure S2.** Sequence alignment of OsURL1, ZmOCL1, and AtGL2.

**Supplemental Figure S3.** Phylogenetic analysis of rice, maize, and Arabidopsis HD-ZIP IV family members.

**Supplemental Figure S4.** Targeted mutagenesi*s* of the *URL1* gene using CRISPR-Cas9 technique.

**Supplemental Figure S5.** The secondary structure in the 3′-UTR of *URL1* predicted by the RNAfold software.

**Supplemental Figure S6.** Characterization of the *Url1* and *URL1* cDNA overexpression lines.

**Supplemental Figure S7.** Tissue-specific expression of *URL1*

**Supplemental Figure S8.** Characterization of the *ROC5* mutant created via CRISPR-Cas9 in the *Url1* mutant background.

**Supplemental Figure S9.** Effects of DEX on the expression of the *URL1* and its target gene *ACL1* in WT

**Supplemental Figure S10.** Phenotypes of abnormal spikelet in the *tpl2/Url1* mutants generated via CRISPR-Cas9.

**Supplemental Figure S11.** ROC5 interacts with the N-Terminus of TPL2.

**Supplemental Figure S12.** Schematic representation of the plant expression vectors *pCAMBIA1305.1-APFHN* and *pCAMBIA1305.1-APFHC*.

**Supplemental Table S1.** Primers of INDEL markers used in map-based cloning.

**Supplemental Table S2.** List of primers used in vector construction.

**Supplemental Table S3.** List of primers used in RT-qPCR and *in situ* hybridization.

**Supplemental Table S4.** List of primers used in EMSA and ChIP.

## Supplementary Material

kiaa121_Supplementary_Data

## Notes

X.L. designed the research; J.F., T.G., Z.X., Y.C., J.Z., L.P., S.A.Z., and S.Y. performed the experiments; J.F., T.G., and X.L. analyzed the data; J.F., T.G., S.A.Z., and X.L. wrote the article.

The author responsible for the distribution of materials integral to the findings presented in this article in accordance with the policy described in the Instructions for Authors (https://academic.oup.com/plphys/pages/general-instructions) is: Xueyong Li (lixueyong@caas.cn)
