# Supplementary material for: The URL1–ROC5–TPL2 transcriptional repressor complex represses the ACL1 gene to modulate leaf rolling in rice
Source: Plant Physiol. 2021 Jan 13;185(4):1722–44. doi: 10.1093/plphys/kiaa121 (PMC8133684; doi:10.1093/plphys/kiaa121)
Supplement: kiaa121_Supplementary_Data [file kiaa121_supplementary_data.docx]

**Supplemental Data**

**Short Title:** URL1/ROC5/TPL2/ACL1 regulation of leaf rolling

**Title:** The URL1-ROC5-TPL2 transcriptional repressor complex represses the *ACL1* gene to modulate leaf rolling in rice

Jingjing Fang,Tingting Guo, Zhiwei Xie, Yan Chun, Jinfeng Zhao, Lixiang Peng, Syed Adeel Zafar, Shoujiang Yuan , Langtao Xiao and Xueyong Li

**
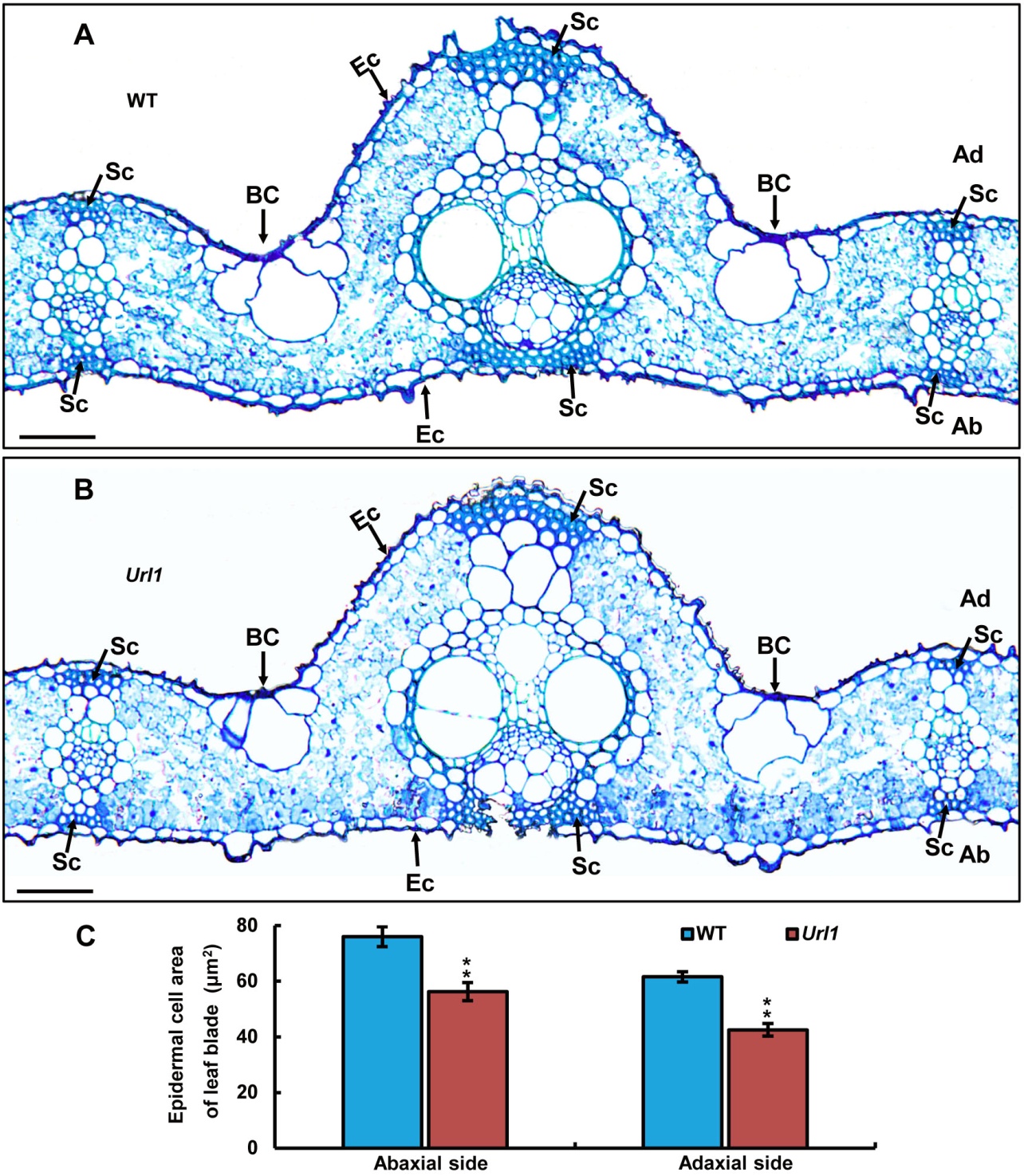
**

**Supplemental Figure S1.** A and B, Transverse section of the Wild-type (WT) and *Url1* mutant mature leaf. Sclerenchymatous cells at the abaxial side showed no difference between the *Url1* mutant and WT. Bars = 50 μm. Ad, Adaxial side; Ab, Abaxial side; Bc, Bulliform cell; Sc, Sclerenchymatous cell. Ec, Epidermal cell (excluding bulliform cell). C, The epidermal cell area of leaf blade in WT and *Url1* at abaxial side and adaxial side, respectively, in transverse view. Data are presented as mean *±* SE (*n* = 8). Significance of data is tested by Student’s *t* test (** *P* < 0.01).

**
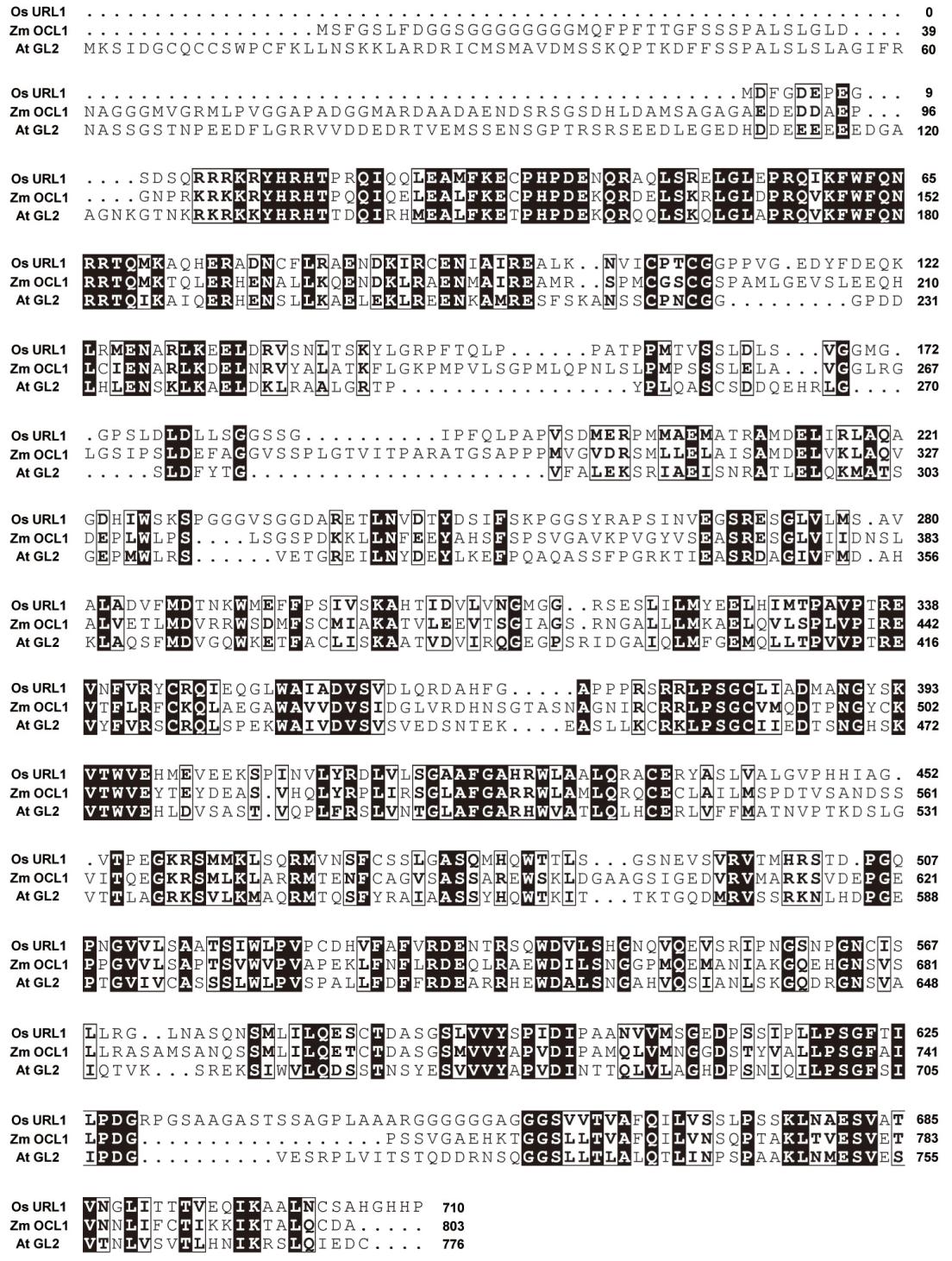
**

**Supplemental Figure S2.** Sequence alignment of OsURL1, ZmOCL1 and AtGL2. The identical residues were in black background while similar residues were boxed. The sequence accession numbers are: OsURL1, MH822135; ZmOCL1, BK008026; AtGL2, AT1G79840.

**
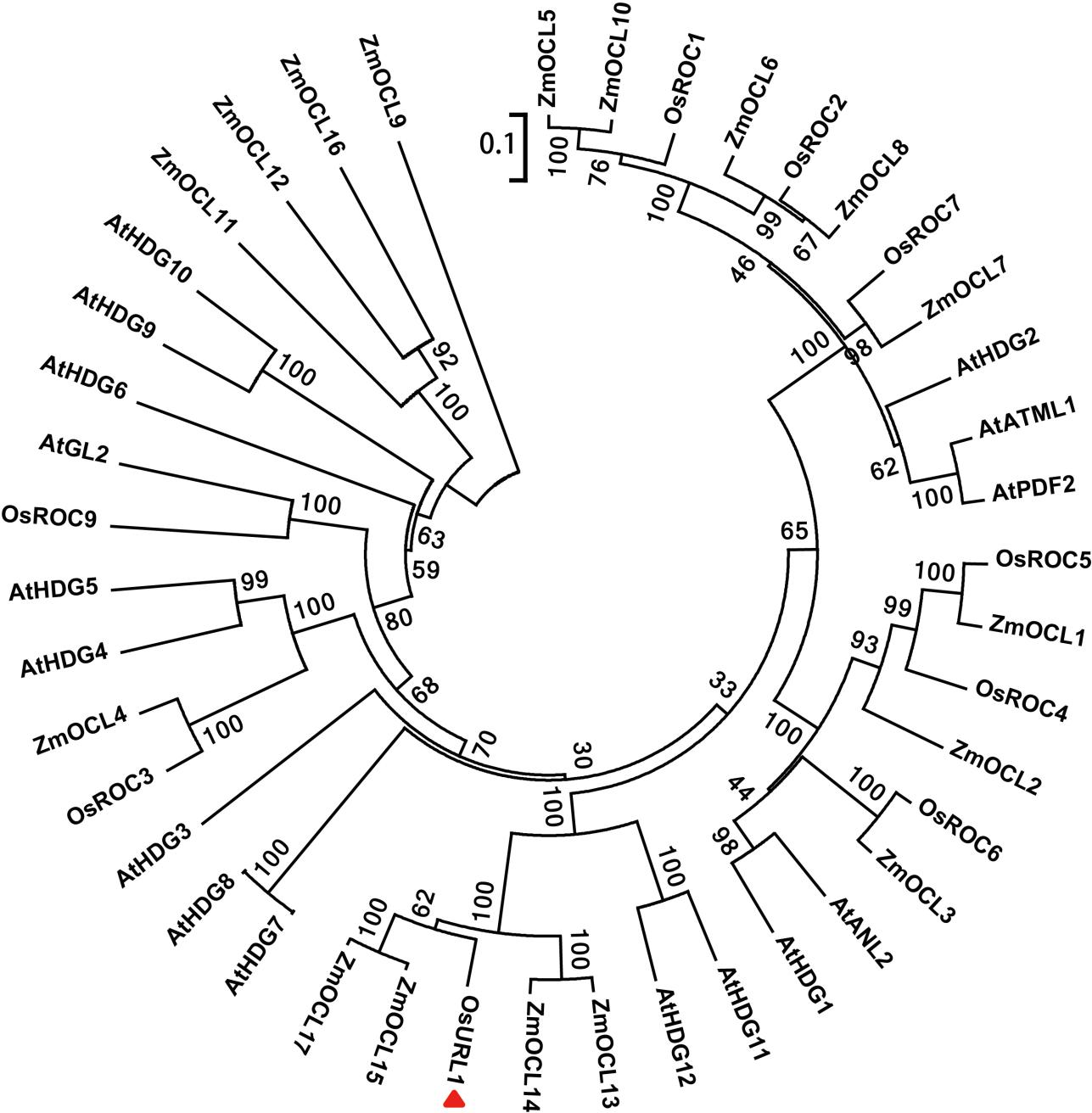
**

**Supplemental Figure S3.** Phylogenetic analysis of rice, maize, and Arabidopsis HD-ZIP IV family members. The sequence accession numbers are: OsROC1, LOC_Os08g08820; OsROC2, LOC_Os04g53540; OsROC3, LOC_Os10g42490; OsROC4, LOC_Os04g48070; OsROC5, [LOC_Os06g35970](http://rice.plantbiology.msu.edu/cgi-bin/ORF_infopage.cgi?orf=LOC_Os06g35970); OsROC6, LOC_Os09g35760; OsROC7, LOC_Os08g04190; OsURL1, MH822135; OsROC9, LOC_Os01g55549; HDG1, [At3g61150](https://www.arabidopsis.org/servlets/TairObject?id=39882&type=locus); HDG2, [At1g05230](https://www.arabidopsis.org/servlets/TairObject?id=137977&type=locus); HDG3, [At2g32370](https://www.arabidopsis.org/servlets/TairObject?id=34983&type=locus); HDG4, [At4g17710](https://www.arabidopsis.org/servlets/TairObject?id=128661&type=locus); HDG5, [At5g46880](https://www.arabidopsis.org/servlets/TairObject?id=134197&type=locus); HDG6, [At4g25530](https://www.arabidopsis.org/servlets/TairObject?id=129045&type=locus); HDG7, [At5g52170](https://www.arabidopsis.org/servlets/TairObject?id=130736&type=locus); HDG8, [At3g03260](https://www.arabidopsis.org/servlets/TairObject?id=39692&type=locus); HDG9, [At5g17320](https://www.arabidopsis.org/servlets/TairObject?id=133706&type=locus); HDG10, [At1g34650](https://www.arabidopsis.org/servlets/TairObject?id=28860&type=locus) ; HDG11, [At1g73360](https://www.arabidopsis.org/servlets/TairObject?id=137927&type=locus); HDG12, [At1g17920](https://www.arabidopsis.org/servlets/TairObject?id=30010&type=locus); ANL2, [At4g00730](https://www.arabidopsis.org/servlets/TairObject?id=128298&type=locus); ATML1, [At4g21750](https://www.arabidopsis.org/servlets/TairObject?id=127172&type=locus); GL2, [At1g79840](https://www.arabidopsis.org/servlets/TairObject?id=28310&type=locus); PDF2, [At3g22480](https://www.arabidopsis.org/servlets/TairObject?id=36086&type=locus); ZmOCL1, BK008026; ZmOCL2, BK008027; ZmOCL3, BK008028; ZmOCL4, BK008029; ZmOCL5, BK008030; ZmOCL6, BK008031; ZmOCL7, BK008032; ZmOCL8, BK008033; ZmOCL9, BK008034; ZmOCL10, BK008035; ZmOCL11, BK008036; ZmOCL12, BK008037; ZmOCL13, BK008038; ZmOCL14, BK008039; ZmOCL15, BK008040; ZmOCL16, BK008041; ZmOCL17, BK008042.

**
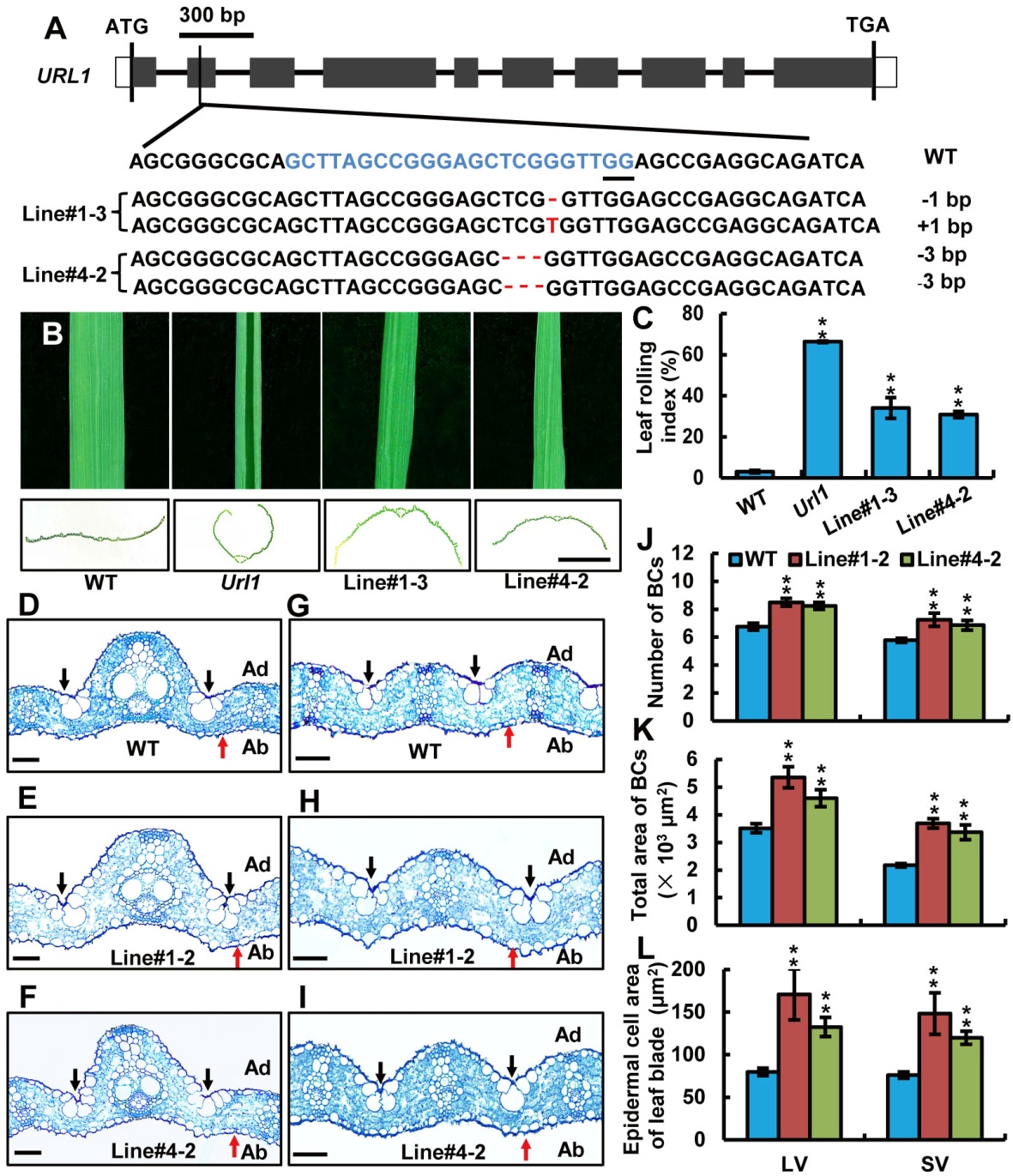
**

**Supplemental Figure S4.** Targeted mutagenesis of the *URL1* gene using CRISPR-Cas9 technique. A, The position of target sequence is shown on the *URL1* gene structure. The spacer sequence is highlighted in blue and the PAM site is underlined. Sequencing result showed the mutation sites highlighted in red. B, Leaf blade (upper pannel) and cross section (lower pannel) of WT, *Url1* mutant and the CRISPR-Cas9 lines. Bars = 1 cm in upper pannel and 0.5 cm in lower pannel. C, Leaf rolling index of WT, *Url1* mutant and the *URL1* CRISPR-Cas9 lines. Data are presented as mean *±* SE (*n* = 15). Significance of data is tested by Student’s *t* test (** *P* < 0.01). D to I, Transverse section of the mature leaf blade of *URL1* CRISPR-Cas9 lines to show the morphological characteristics of bulliform cells near large veins (D-F) and small veins (G-I). Black arrows indcate the bulliform cells. Red arrows indicate the epidermal cells. Ad, adaxial; Ab, abaxial. Bars = 50 μm. J and K, Bulliform cell numbers (J) and area (K) of WT, *Url1* mutant and *URL1* CRISPR-Cas9 lines. Data are presented as mean *±* SE (*n* = 8). Significance of data is tested by Student’s *t* test (** *P* < 0.01). L, Epidermal cell area of WT, *Url1* and URL1 CRISPR-Cas9 lines at the abaxial side. Data are presented as mean *±* SE (*n* = 8). Significance of data is tested by Student’s *t* test (** *P* < 0.01).

**
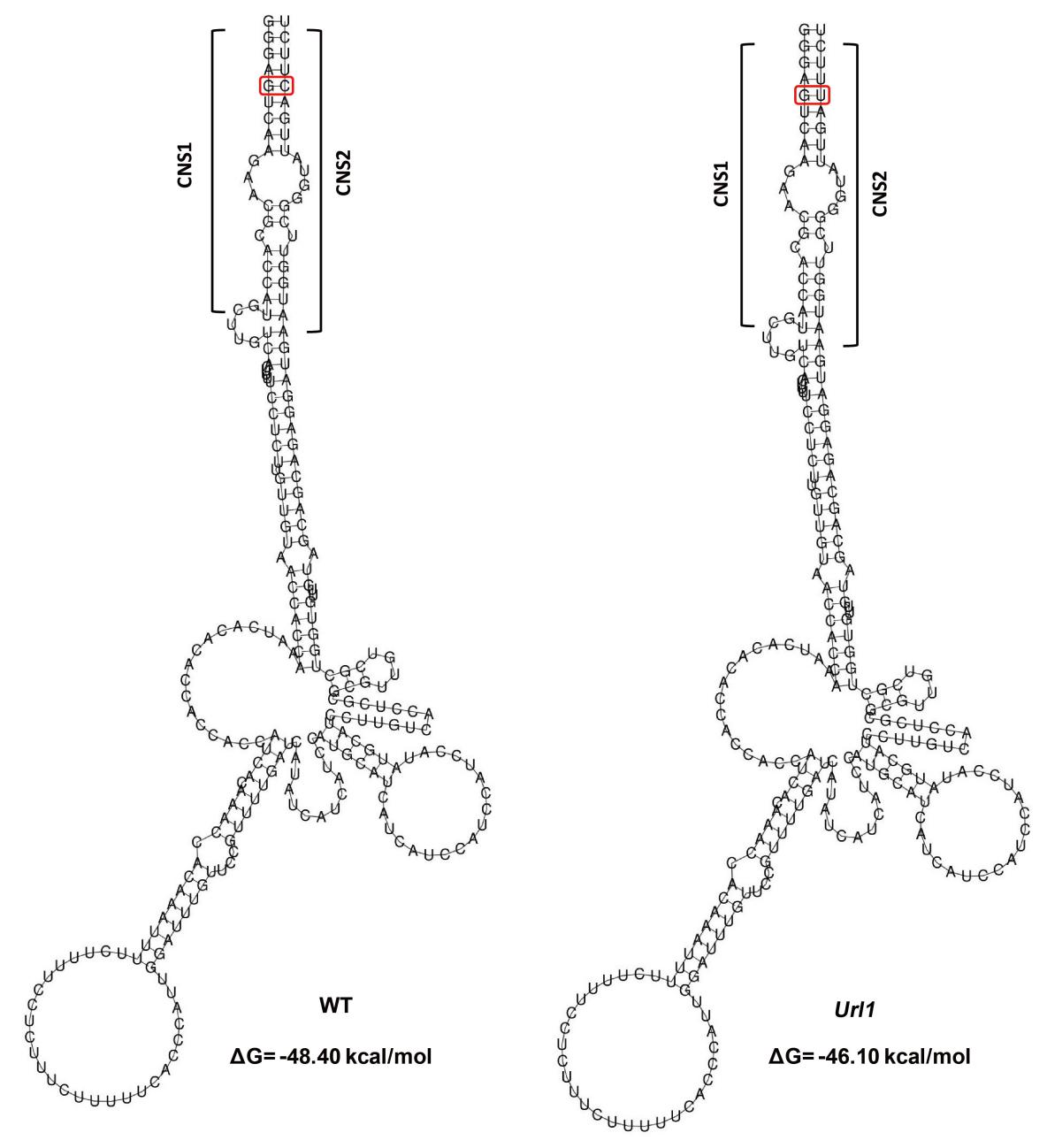
**

**Supplemental Figure S5.** The secondary structure in the 3′-UTR of *URL1* predicted by the RNAfold software. Prediction of base pairing between the conserved 19 nt CNS1 and 21 nt CNS2 motifs in the 3'-UTR of WT (left) and mutant (right) *URL1* genes. The C679T substitution in the *Url1* mutant changed the G-C base pairing to G-U mismatch (highlighted in red box). The ΔG value indicates the free energy of the two predicted second structure.


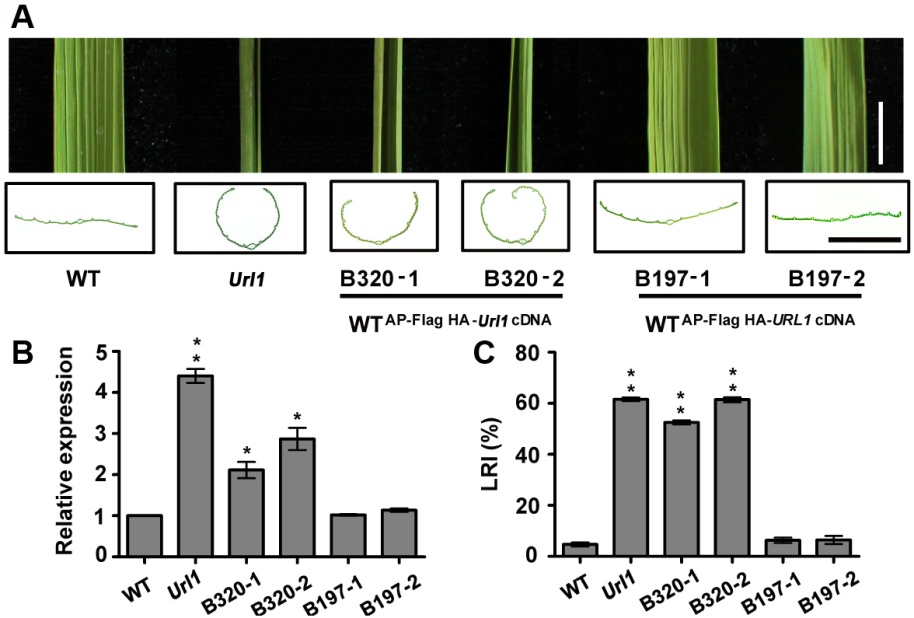


**Supplemental Figure S6.** Characterization of the *Url1* and *URL1* cDNA overexpression lines. A, Leaf phenotype of transgenic plants overexpressing the *Url1* mutant cDNA (B320) or the WT *URL1* cDNA (B197) in the WT background. B, RT-qPCR analysis of the *URL1* transcript level in cDNA overexpression lines. Data are presented as mean ± SE (*n* = 3). Significance of data is tested by Student’s *t t*est (* *P* < 0.05, ** *P* < 0.01). C, Leaf rolling index of cDNA overexpression lines. Data are presented as mean ± SE (*n* = 15)*.* Significance of data is tested by Student’s *t* test (** *P* < 0.01).


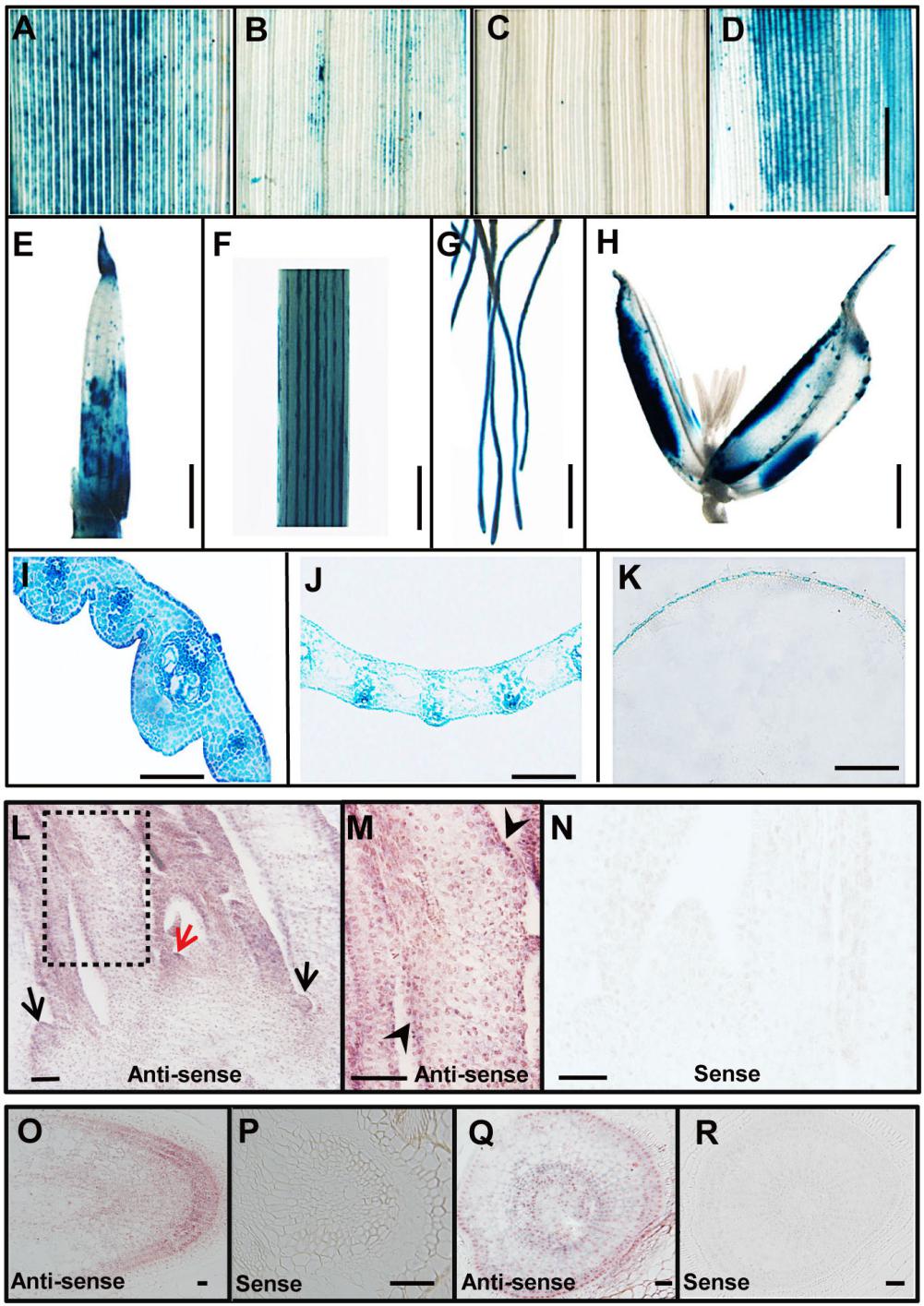


**Supplemental Figure S7.** Tissue-specific expression of *URL1.* A to H, Expression pattern of GUS gene driven by the *URL1* promoter. Expression of *URL1* in the young (A), mature (B) and old (C) leaf blade, leaf sheath (D), tiller bud (E), stem (F), root (G) and glume (H) was analyzed. Bars = 2.5 mm in (A-D), 1 mm in (E-G), and 5 mm in (G). I to K, Cross section of GUS stained young leaf blade (I), sheath (J) and root (K). Bars = 200 μm. L to M, *In situ* hybridization to detect *URL1* transcripts on the longitudinal section of shoot base. The boxed image in (L) was magnified in (M). The black arrows in (L) indicate the axillary meristems. The red arrow in (L) indicates the leaf primordium. The black arrowheads in (M) indicate the *in situ* hybridization signals of epidermal cells in longitudinal direction. Bars = 50 μm. N to Q, *In situ* hybridization to detect *URL1* transcripts on the longitudinal (N, O) and cross (P, Q) section of crown root primordial in WT of 7-d-old rice seedling. Bars = 50 μm.

**
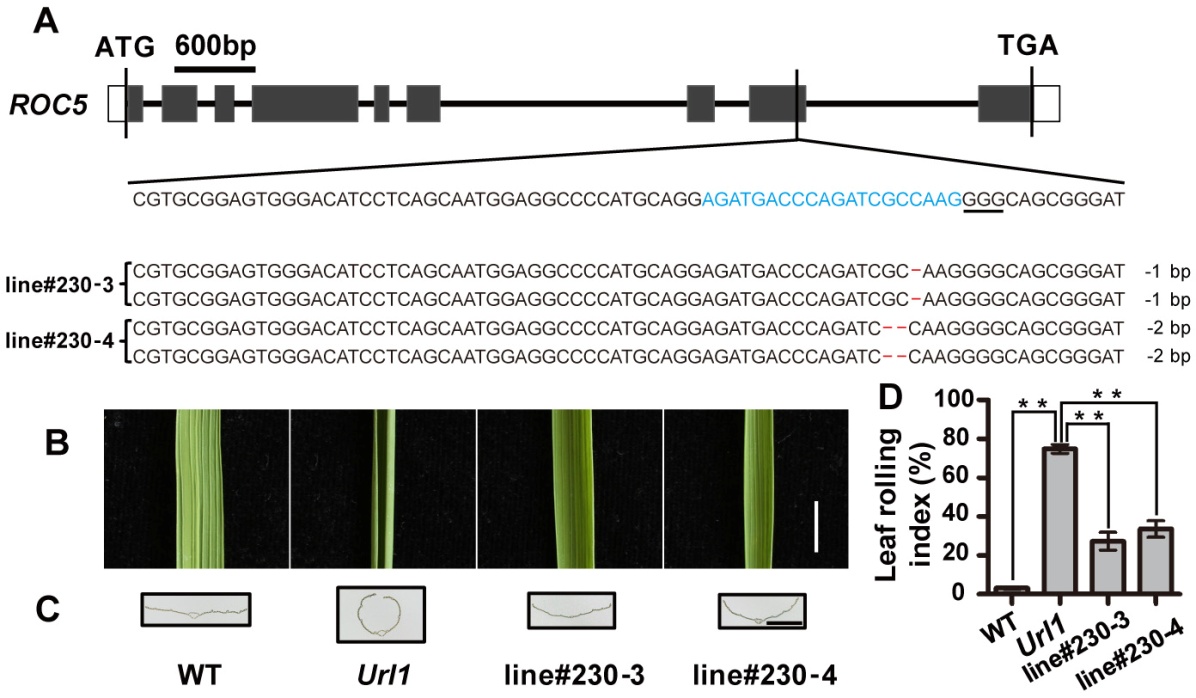
**

**Supplemental Figure S8.** Characterization of the *ROC5* mutant created via CRISPR-Cas9 in the *Url1* mutant background. A, Targeted mutagenesis of the *ROC5* gene in the *Url1* mutant using CRISPR-Cas9 system. The position of target site is given on the *ROC5* gene structure. The spacer sequence is highlighted in blue and the PAM site is underlined. The deleted nucleotides are indicated in red dash lines. B and C, Leaf blade (B) and cross section (C) of WT, *Url1* and *ROC5* CRISPR-Cas9 lines. Bars = 0.5 cm in (B) and 0.25 cm in (C). D, Leaf rolling index of WT, *Url1* and *ROC5* CRISPR-Cas9 lines. Data are presented as mean ± SE (*n* = 15). Significance of data is tested by Student’s *t* test (** *P* < 0.01).


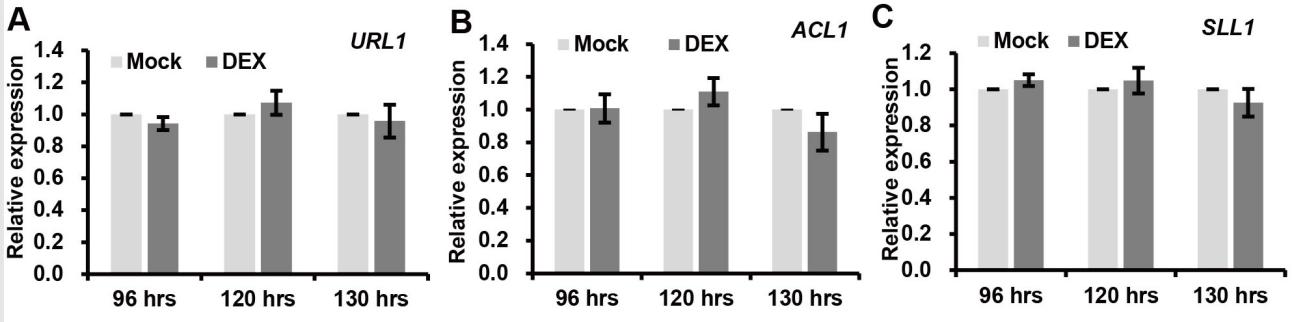
**Supplemental Figure S9.** Effects of DEX on the expression of the *URL1* and its target gene *ACL1* in WT*.* A to C, RT-qPCR analysis of *URL1* (A) and *ACL1* (B) expression in WT calli treated with 30 µM DEX or Mock for different time. The *SLL1* gene was used as a negative control (C). Data are presented as mean ± SE (*n* = 3).


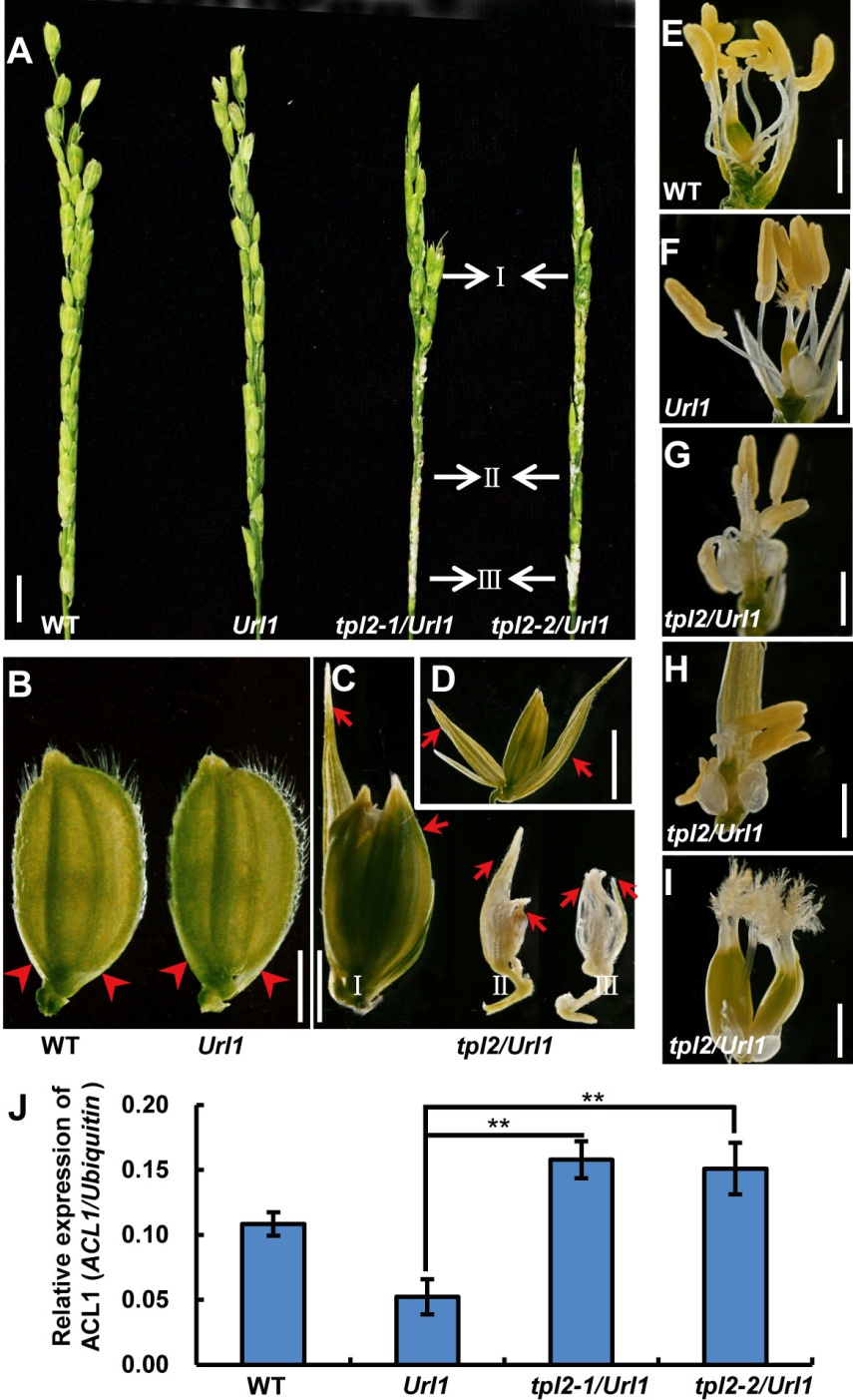


**Supplemental Figure S10.** Phenotypes of abnormal spikelet in the *tpl2/Url1* mutants generated via CRISPR-Cas9. A, Panicle morphology of WT, *Url1* and *tpl2/Url1*. Bar = 1 cm. B to D, Types of the spikelet hulls in WT, *Url1* and *tpl2/Url1*. The spikelet hull of typeⅠin (C) was dissected and viewed in (D). The positions of spikelet hulls of type I, II, III were pointed with arrows in (A). Sterile lemma in WT and *Url1* mutant are short and inconspicuous (red arrowheads), whereas the spikelet in *tpl2/Url1* mutants shows overgrowth of sterile lemma (red arrows) that resemble paleae and lemmas. Bars = 2 mm in (B, C), and 5 mm in (D). E to I, Types of the flower organs in WT, *Url1* and *tpl2/Url1* spikelets after removing the lemma and palea. The number of stamen was reduced in the *tpl2/Url1* mutant (G-I). Bars = 1 mm. J, RT-qPCR analysis of *ACL1* expression in WT, *Url1* and *tpl2/Ur1l.* Data are presented as mean ± SE (*n* = 3). Significance of data is tested by Student’s *t* test (** *P* < 0.01).


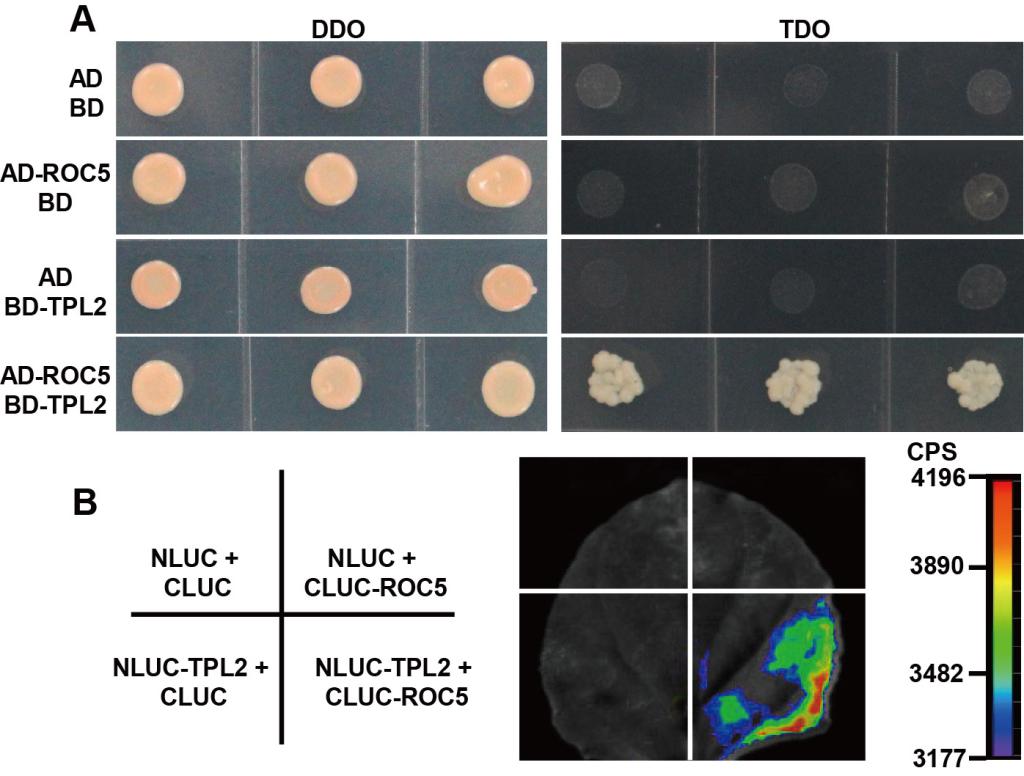


**Supplemental Figure S11.** ROC5 interacts with the N-Terminus of TPL2. A, ROC5 interacts with the N-terminus of TPL2 in the yeast two-hybrid assay. Transformed yeasts were spotted on SD-Leu-Trp (DDO) or SD-Leu-Trp-His (TDO) medium. The empty vectors were used as controls. B, ROC5 interacts with the N-terminus of TPL2 *in vivo* as indicated by the LCI assay.


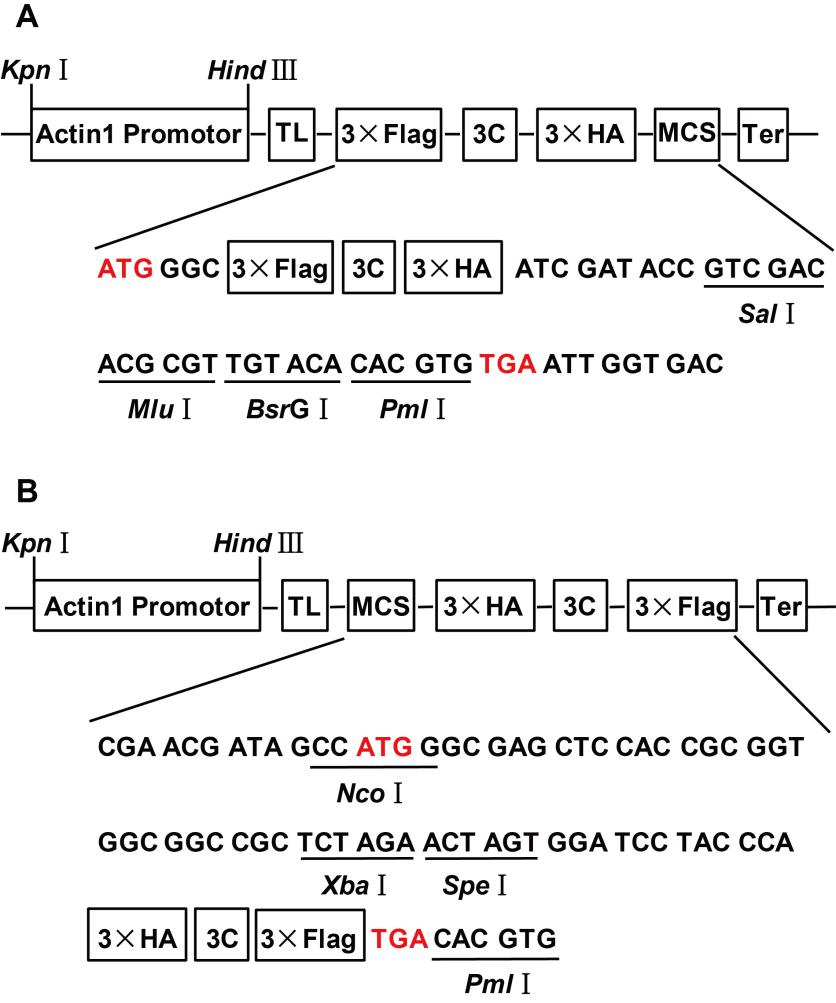


**Supplemental Figure S12.** Schematic representation of the plant expression vectors. *pCAMBIA1305.1-APFHN* and *pCAMBIA1305.1-APFHC*. A, *pCAMBIA1305.1-APFHN* in which target gene can be cloned in the multiple cloning sites (MCS) after the N-terminal Flag and HA tags. B, *pCAMBIA1305.1-APFHC* in which target gene can be cloned in the MCS in front of the C-terminal Flag and HA tags. The translation start codon ATG and stop codon TGA are highlighted in red. TL: translational leader, 5’-UTR of Tobacco Etch virus; 3C: 3C protease cleavage site; 3 x Flag: three tandem copies of Flag tag; 3 x HA: three tandem copies of HA tag.

Supplemental Table S1. Primers of INDEL markers used in map-based cloning

| Primer name | Sequence |
| --- | --- |
| M1-F | 5’-CACCTTTTGTCTAATCAAATCAGTTT-3’ |
| M1-R | 5’-TCTTTGGTGCTGAAAGTATGTAAGA-3’ |
| M2-F | 5’-GCAACCACGAAGAACTGTAG-3’ |
| M2-R | 5’-CCATTCGCACGACTCTAGAC-3’ |
| M3-F | 5’-GGTTCTTCCAGTAAGGTGAT-3’ |
| M3-R | 5’-AAAGACACTCCTCAGATGCG-3’ |
| M4-F | 5’-CGTATGCACGCCATATCCTA-3’ |
| M4-R | 5’-ACACGACCTTGGCAACAAAC-3’ |
| M5-F | 5’-TCAGATCCCCCAGACTGAAA-3’ |
| M5-R | 5’-TATCAGACTACTCCCTCCGT-3’ |
| M6-F | 5’-GCACAGTTCGACAAGAGCAA-3’ |
| M6-R | 5’-CCTCCGTTTCACAGTGTAAG-3’ |

Supplemental Table S2. List of primers used in vector construction

| Primer name | Sequence |
| --- | --- |
| URL1-gDNA-F1 | 5’-TGCAGGCATGCAAGCTTCCTCTTTGCTCTATTTGTACG-3’ |
| URL1-gDNA-R1 | 5’-CGTTCTCCATGCGAAGCTTCTGCTCGTCGAAGTAGTC-3’ |
| URL1-gDNA-F2 | 5’-TGCAGGCATGCAAGCTTCGCATGGAGAACGCC-3’ |
| URL1-gDNA-R2 | 5’-TCACCAATTCACACGTGTCACACATATCACCGTTCAG-3’ |
| URL1CDSPP F | 5’-ACGCGTTGTACACACGTGATGGATTTCGGCGACGAACCC-3’ |
| URL1CDSPP R | 5’-GTCACCAATTCACACGTGCCATTTATAACCGAAATGAATG-3’ |
| URL1-RNAi-F1 | 5’-CTTCTGCACTAGGTACCATCACCACCACCGTCGAGCA-3’ |
| URL1-RNAi-R1 | 5’-AGCTCAGGCCTGGTACCTGGAGTCCATGAATGCGAGC-3’ |
| URL1-RNAi-F2 | 5’-AGAATTCCCGGGGATCCATCACCACCACCGTCGAGCA-3’ |
| URL1-RNAi-R2 | 5’-ACGTAGTCGACGGATCCTGGAGTCCATGAATGCGAGC-3’ |
| URL1T1F | 5’-GTTGCTTAGCCGGGAGCTCGGGT-3’ |
| URL1T1R | 5’-AAACACCCGAGCTCCCGGCTAAG-3’ |
| ROC5T1F | 5’-GGCAGATGACCCAGATCGCCAAG-3’ |
| ROC5T1R | 5’-AAACCTTGGCGATCTGGGTCATC-3’ |
| UF | 5’-CTCCGTTTTACCTGTGGAATCG-3’ |
| gR-R | 5’-CGGAGGAAAATTCCATCCAC-3’ |
| Pps-R | 5’-TTCAGAGGTCTCTACCGACTAGTATGGAATCGGCAGCAAAGG-3’ |
| Pgs-L | 5’-AGCGTGGGTCTCGCTCGACGCGTATCCATCCACTCCAAGCTC-3’ |
| attB1-URL1F | 5’-GGGGACAAGTTTGTACAAAAAAGCAGGCTCAATG GATTTCGGCGAC GAACCCGAG-3’ |
| attB1-URL1R | 5’-GGGGACCACTTTGTACAAGAAAGCTGGGTCTGAAGG CTTTACATTAATGGACAG-3’ |
| URL1CDSSSF | 5’-GACACGTGTAACTAGTATGGATTTCGGCGACGAACCC-3’ |
| URL1CDSSSR | 5’-CCTGCAGGTAACTAGTTGAAGGCTTTACATTAATGGA CAG-3 |
| URL1PromKNF | 5’-ACGAATTCGAGCTCGGTACCGCAAGACACAAGATG TGTAGATCG-3’ |
| URL1PromKNR | 5’-CTCAGATCTACCATGGGTTCGTCGCCGAAATCCAT-3’ |
| ACL1gDNAHPF | 5’-TTGTAGGTAGAAGCTTCAGGCTCACTGTTGGGAGATTGCC-3’ |
| ACL1gDNAHPR | 5’-GTCACCAATTCACACGTGTGCGTCACACAGCACGTATTTCAG-3’ |
| VP16-URL1BBF1 | 5’-TGGACGATTTCGATCTCGACATGCTGGGTTCTATGGAT TTCGGCGACGAACCC-3’ |
| VP16-URL1BBF2 | 5’-TGTATCGCCGAGATCTATGGACGCGCTGGACGATTTCGATCTCGACA-3’ |
| VP16-URL1BBR | 5’-TAGACTAGGTGGATCCTCAGGGGTGGTGGCCATGGGC-3’ |
| BD ROC5 NS F | 5’-AGGAGGACCTGCATATGGATCCAAGGAAGAGGACTTTCTCG-3’ |
| BD ROC5 NS R | 5’-GCCGCTGCAGTCGACCGTTCTTGACTCAGGCTCGTC-3’ |
| BD URL1 ES F | 5’-CATGGAGGCCGAATTCATGGATTTCGGCGACGAACCC-3’ |
| BD URL1 ES R | 5’-GCCGCTGCAGGTCGACTCAGGGGTGGTGGCCATGGGC-3’ |
| AD ROC5 EB F | 5’-ATGGAGGCCAGTGAATTCATCCAAGGAAGAGGACTTTC-3’ |
| AD ROC5 EB R | 5’-CTCGAGCTCGATGGATCCCGTTCTTGACTCAGGCTCGT-3’ |
| AD URL1 EB F | 5’-ATGGAGGCCAGTGAATTCATGGATTTCGGCGACGAACC-3’ |
| AD URL1 EB R | 5’-CTCGAGCTCGATGGATCCTCAGGGGTGGTGGCCATGGG-3’ |
| BD TPL2 ES F | 5’-GCCATGGAGGCCGAATTCGTCGTCGCTTAGCAGGGA-3’ |
| BD TPL2 ES R | 5’-TATGGGATGCTCAGACAGGAGTCGACCTGCAGCGGCCG-3’ |
| TPL2N-nluc F | 5’-GCTCGAGTAGTCGACATGTCGTCGCTTAGCAGGGAG-3’ |
| TPL2N-nluc R | 5’-CGAGATCTGGTCGACTCCTGTCTGAGCATCCCATAC-3’ |
| ROC5-nluc F | 5’-GCTCGAGTAGTCGACATGAGCTTTGGGGGCCTCTTTGAC-3’ |
| ROC5-nluc R | 5’-CGAGATCTGGTCGACGGCGTCGCACTGCAGCGCCGTC-3’ |
| cluc-ROC5 F | 5’-TCCCGGGGCGGTACCATGAGCTTTGGGGGCCTCTTTGAC-3’ |
| cluc-ROC5 R | 5’-GCTCTGCAGGTCGACTCAGGCGTCGCACTGCAGCGCCGTC-3’ |
| cluc-ROC8 F | 5’-TCCCGGGGCGGTACCATGGATTTCGGCGACGAACC-3’ |
| cluc-ROC8 R | 5’-GCTCTGCAGGTCGACTCAGGGGTGGTGGCCATGGG-3’ |
| ROC8-nluc(F) | 5’-GCTCGAGTAGTCGACATGGATTTCGGCGACGAACC-3’ |
| ROC8-nluc(R) | 5’-CGAGATCTGGTCGACGGGGTGGTGGCCATGGGCGGAG-3’ |
| TPL2(CRISPR)F | 5’-GGCAATATGACTGTTTGGGCTCAA-3’ |
| TPL2(CRISPR)R | 5’-AAACTTGAGCCCAAACAGTCATA-3’ |

Supplemental Table S3. List of primers used in RT-qPCR and *in situ* hybridization

| Primer name | Sequence |
| --- | --- |
| (URL1-III)URL1-RT-F | 5’-CAATCAGTTTTCAGGACATCACC-3’ |
| (URL1-III)URL1-RT-R | 5’-CATTGAAGGCTTTACATTAATGGAC-3’ |
| URL1-I F | 5’-GCAGATGCACCAGTGGACGAC-3 |
| URL1-I R | 5’-GAGGTGGCGGCGCTGAGGAC-3 |
| URL1-II F | 5’-GAGTTGGTTGGCGCGTTGAATG-3 |
| URL1-II R | 5’-GGATCATGGCTAGCTGGATAATGGATG-3 |
| URL1(NRO)F1 | 5’-CGTGTGTTTGTTTCCCGTGTTGTG-3’ |
| URL1(NRO)R1 | 5’-CTTGATCTGCCTCGGCTCCAAC-3’ |
| URL1(NRO)F4 | 5’-GTTCGACGCCACAACCATTCTCC-3’ |
| URL1(NRO)R4 | 5’-CTTTGGACACGATGCTTGGGAAG-3’ |
| URL1(NRO)F5 | 5’-CCATTTTGATCTTGTGATGTTTGTGTTG-3’ |
| URL1(NRO)R5 | 5’-GTCATGATGTGCAGCTCCTCGTAC-3’ |
| Actin(NRO)F | 5’-CTGCTATGTACGTCGCCATC-3’ |
| Actin(NRO)R | 5’-GATTGCAGAACTAAATCTCATCTACATG-3’ |
| ACL1-RT-F | 5’-CTGAAGCTGAACCTCTCGCTG-3’ |
| ACL1-RT-R | 5’-GGAGCATGACGTAGATGAAGCAG-3’ |
| SRL1-RT-F | 5’-GTTTGTCCACTAAGCTTTCCAGAC-3’ |
| SRL1-RT-R | 5’-GACATTCATCTTTTGCAACTTGTC-3’ |
| RL14- RT-F | 5’-CTCTTTCAGGCATTCCATTGATG-3’ |
| RL14- RT-R | 5’-CAACACCTTGTCAGCTTTCAAGC-3’ |
| ZHD1- RT-F | 5’-CGGACCCCGGTATGGTAG-3’ |
| ZHD1- RT-R | 5’-CGAGAACGAATGCTCTCTCAG-3’ |
| ADL1- RT-F | 5’-ATCTGGCTTCTTCTTTGGGG-3’ |
| ADL1- RT-R | 5’-GGTTTTCGAAGGAAGGGGCA-3’ |
| SLL1-RT-F | 5’-CAGGTGTCCAACCATGAGC-3’ |
| SLL1-RT-R | 5’-GCCTCTGTGATTGCCATCTAAT-3’ |
| Ubiquitin -RT-F | 5’-AACCAGCTGAGGCCCAAGA-3’ |
| Ubiquitin-RT- R | 5’-ACGATTGATTTAACCAGTCCATGA-3’ |
| LUC qRT F | 5’-GTCCTATGATTATGTCCGGTTATGT-3’ |
| LUC qRT R | 5’-GTCTTCGTCCCAGTAAGCTATG-3’ |
| RLUC qRT F | 5’-TGATAACTGGTCCGCAGTGG-3’ |
| RLUC qRT R | 5’-TAATACACCGCGCTACTGGC-3’ |
| URL1(situ)F | 5’-CTTGCATGCCTGCAGCTTTCGCCCATCCATTATCC-3’ |
| URL1(situ)R | 5’-TTCGAGCTCGGTACCAAGCATGGTGCGTTCTTGAC-3’ |

Supplemental Table S4. List of Primers Used in EMSA and ChIP

| Primer name | Sequence |
| --- | --- |
| ACL1 L1-BOX I | 5’-CCGACTAATGGCAATATATACGTGCAAAGA**TACATTTA**TATACCATATCCTGTACTAAGCAATAACCTA-3’ |
| ACL1 L1-BOX II | 5’-TTATCATAAAAGAAACTATAATATTAACTTA**TACATTTA**CATATTTATACTAAATTTTTAAATAAGGCAA-3’ |
| mACL1 L1-BOX I | 5’-ACCGACTAATGGCAATATAAAGATATACCATATCCTGTACTAAGCAATAACCTA-3’ |
| mACL1 L1-BOX II | 5’-TTATCATAAAAGAAACTATAATATTAACTTACATTACTTTAAGGCAA-3’ |
| ACL1- I (CHIP)F | 5’-ATCATTTATTTTGTTGTGTGTTTTATCAT-3’ |
| ACL1- I (CHIP)R | 5’-GTACCCTCTGTCCAATATATGATGTC-3’ |
| ACL1- II (CHIP)F | 5’-CACCATGGAATTAAAGAAGAGCAC-3’ |
| ACL1- II (CHIP)R | 5’-GTGCCACTAGGTTATTGCTTAGTAC-3’ |
| ACL1-Ⅲ(CHIP)F | 5’-CATAATTACCCAATAAGTCCAGCTAAC-3’ |
| ACL1-Ⅲ(CHIP)R | 5’-GTGAGCCTGGTTGAAAATTGTAAC-3’ |
| Ubiquitin F | 5’-AACCAGCTGAGGCCCAAGA-3’ |
| Ubiquitin F | 5’-ACGATTGATTTAACCAGTCCATGA-3’ |
